# A Spatiotemporal Hybrid Integration Algorithm for Airborne Target Detection and Localization in Multi-Illuminator GNSS-Based Passive Radar

**DOI:** 10.3390/s26206417

**Published:** 2026-10-11

**Authors:** Fan Xu, Feng Luo, Zixuan He, Jiahao Han, Wanyao Wu, Linrang Zhang

**Affiliations:** National Laboratory of Radar Signal Processing, Xidian University, Xi’an 710071, China; 21021110339@stu.xidian.edu.cn (F.X.); 21021110329@stu.xidian.edu.cn (Z.H.); 22021110282@stu.xidian.edu.cn (J.H.); 24021110045@stu.xidian.edu.cn (W.W.); lrzhang@xidian.edu.cn (L.Z.)

**Keywords:** spatiotemporal hybrid integration, target localization, multistatic radar, joint detection and localization

## Abstract

**Highlights:**

**What are the main findings?**

- Existing methods require a four-dimensional search over target azimuth, elevation, and their rates. Errors in the searched parameters propagate into the Doppler frequency rate (DFR) calculation, degrading cross-link alignment and integration performance.
- The unified kinematic vector (UKV) provides a link-decoupled representation of the target’s geometrical features. It provides common geometrical constraints for link-wise coherent integration, interlink range scaling alignment (IRSA), detection, and localization, thereby avoiding direction-of-arrival (DOA) rate estimation and the associated error propagation.

**What are the implications of the main findings?**

- By exploiting multiple global navigation satellite system (GNSS) illuminators as spatial integration resources, UKV–IRSA extends the available integration gain beyond the single-illuminator limit and offers broad application potential for GNSS-based passive radar systems.
- The multi-illuminator approach permits joint aircraft detection and localization using only a single passive receiver, enabling the prospect of highly mobile, covert, and precision air surveillance that requires no dedicated transmit infrastructure.

**Abstract:**

This paper addresses the fundamental power-budget limitation of global navigation satellite system (GNSS)-based passive radar in aircraft detection and localization by exploiting multiple satellite illuminators for spatiotemporal hybrid integration. In contrast to single illuminator operation, cross-link coupling effects, manifested as disparate range and Doppler signatures across individual bistatic links, pose a formidable barrier to coherent energy combination. To surmount this challenge, we first introduce a unified kinematic vector (UKV) that projects the target state into a link decoupled common domain. The UKV encapsulates the target direction of arrival (DOA) and monostatic range, three parameters that remain approximately invariant across all bistatic links sharing a common receiver. Building upon the UKV, we develop an interlink range scaling alignment (IRSA) algorithm that coherently integrates target returns along a UKV-constrained trajectory and project the accumulated energy onto a shared monostatic-range, zero-Doppler grid. This registration aligns target responses from disparate links at a unified spatial coordinate, thereby enabling genuine signal-level fusion across illuminators. When applied across multiple coherent frames, UKV–IRSA elevates sub-noise target energy to clearly detectable levels. Extensive numerical simulations and real-data experiments corroborate the efficacy of the proposed approach.

## 1. Introduction

The repurposing of Global Navigation Satellite System (GNSS) transmissions as signals of opportunity (SoOPs) has garnered escalating research attention over the past two decades, encompassing a diverse array of constellations including the Global Positioning System (GPS) [1], BeiDou [2], Globalnaya Navigatsionnaya Sputnikovaya Sistema (GLONASS) and Galileo [3]. One prominent branch of this field, termed GNSS reflectometry, has found widespread application across the remote sensing landscape, encompassing ocean state estimation, sea-level altimetry, soil moisture retrieval, vegetation biomass assessment, and ice-sheet monitoring [4,5,6,7,8].

GNSS-based passive bistatic radar (PBR) is another important application in object surveillance. Although passive radar is a conventional radar system, the emergence of new generations of GNSS satellites has facilitated its advancement. For example, exploiting the forward scattering effect [9,10,11], the target’s radar cross section (RCS) grows rapidly near the PBR baseline. This makes PBR equipped with high-orbit GNSS transmitters suitable for aircraft detection [12], especially for stealth aircraft and uncrewed aerial vehicles (UAV). Moreover, benefiting from the global coverage of GNSS, PBR can be deployed anywhere, even at the poles. Recent advances in multistatic radar deployment optimization [13] have shown that radar coverage can be significantly improved through careful placement of multiple nodes. Therefore, GNSS-based PBR is emerging as a passive surveillance technique with low size, weight, and power requirements and global deployment capability.

However, the main bottleneck of the GNSS-based PBR application is the excessively low signal power density reaching the ground (−135 dBW/$m^{2}$) [14]. As a result, the reflected target echoes are usually far below the background noise and interference, which severely constrains the detection performance of PBR. To address this limitation, the prevailing strategy involves enhancing the target signal-to-noise ratio (SNR) level through long-duration coherent integration (tens of seconds). In [15,16], a long-time hybrid integration algorithm is proposed to segment the entire signal into multi-frames, where the target can be accumulated through intra-frame coherent integration and inter-frame non-coherent integration. To properly remove the intra-frame target motion, ref. [17] utilizes a long-time hybrid integration algorithm to address the large range cell migration (RCM) and time-varying Doppler frequency modulation (DFM) problems, where the RCM is corrected by the second-order keystone transform (SKT) and modified scaled Fourier transform (MSCFT), and the DFM is compensated with a novel range frequency reversal process, then, a spatial projection method is adopted to integrate the moving target energy in space dimension by dividing the detection area into 3D grids. Ref. [18] exploits a modified Radon Fourier transform (RFT) to correct range and Doppler migration; the real-data experiments therein confirm that motion compensation and long-time integration improve the SNR and extend the detection range. In [19], the odd phase terms of the target signal are compensated via the range-symmetry transform, and the target is mapped in Doppler centroid (DC) and Doppler frequency rate (DFR) domain, but the valuable range information is abandoned, which obstructs subsequent target localization. Beyond SNR enhancement, effective clutter suppression is also critical for passive radar systems, as demonstrated by the low-complexity extensive cancellation algorithm-batches and subbands (ECA-BS) algorithm in [20]. Although such methods are effective for accumulating target energy over the time domain, the accumulated energy is inherently limited by the total energy radiated by one single satellite during the integration period, which constrains their performance. Moreover, the coherent integration time is limited by target motion and RCS fluctuation, which fundamentally constrains the performance of these approaches.

In recent years, an alternative paradigm leveraging multiple GNSS transmitters for maritime moving target (MMT) detection has emerged [2,21,22,23,24]. This approach capitalizes on the geometric reality that typically more than four satellites from a single GNSS constellation are simultaneously visible from any point on the Earth’s surface [25,26]. Maritime targets have been the primary focus of these studies owing to their large RCS, relatively low velocities, and negligible altitude above sea level—characteristics that substantially simplify both experimental validation and algorithmic design. In [21], multiple range-Doppler (RD) maps corresponding to echoes from individual GNSS satellites are first generated via time migration compensation (TMC). Building upon this foundation, ref. [22] reinterprets TMC as a special case of joint space-time compensation and proposes a generalized multistatic compensation (MC) technique. Following MC, target responses in the RD domain are projected onto the direction-of-arrival (DOA) and its temporal derivative domain, where their energy can be aligned for noncoherent accumulation across bistatic links. However, the resulting localization accuracy is acutely sensitive to the precision of DOA and DOA-rate estimates, particularly for long-range, high-velocity targets. In [2], a space-time hybrid integration scheme is introduced to alleviate the computational burden of the MC step. Target energy is first integrated in the range-DFR domain via prolonged coherent integration; subsequently, multiple range-DFR maps are projected onto an *x*-*y*-*v* domain representing the surveillance area and the target’s tangential velocity component, thereby achieving passive multistatic radar (PMR) echo combination. While this approach effectively reduces computational complexity, it hinges on the assumption that the estimated tangential velocity is orthogonal to the target position vectora condition rarely satisfied in practice. Moreover, the localization pipeline depends on high-resolution DFR measurements, which demand coherent integration times exceeding one minute to achieve sub-0.01-Hz/s resolution [27,28,29]; such durations are entirely incompatible with the decorrelation interval of airborne targets [30,31]. More recently, Ragel et al. [32] demonstrated multistatic data fusion for aircraft via common-domain parameter search combined with chirp Z-transform (CZT)-based compensation, termed common-domain chirp Z-transform (CD-CZT). Nevertheless, its requirement for multi-dimensional search over azimuth, elevation, and their respective rates incurs prohibitive computational overhead. Furthermore, its Doppler-rescaling factor is governed by as many as eight geometric and kinematic parameters, whose estimation errors accumulate and destabilize cross-link Doppler alignment, thereby undermining its practical engineering viability. In summary, conventional MMT detection techniques universally disregard target altitude information and thus constitute a degenerate special case of the more general aerial target detection problem. Consequently, they cannot be directly transplanted to airborne surveillance scenarios. Additionally, these methods exhibit pronounced sensitivity to specific parameter measurements-most notably the DOA rate and DFR-compromising overall system robustness.

In this paper, a multi-GNSS interlink data fusion method based on a unified kinematic vector (UKV) and interlink range scaling alignment (IRSA), termed UKV–IRSA, is proposed for the joint detection and localization of airborne moving targets in GNSS-based passive multistatic radar. The target is described by the UKV, comprising the DOA and the Monostatic range, that is invariant across all bistatic links. Driven by each UKV hypothesis, the bistatic range trajectory of every transmitter–receiver link is fixed by the multistatic geometry, so that the RFT is carried out under a common geometric constraint instead of independently per link, and the link-dependent DC is estimated during coherent integration. The integrated bistatic range–Doppler (BRD) responses are then aligned to a common monostatic range–Doppler (MRD) domain by IRSA, realized with the CZT, in which only the range axis is rescaled. A Doppler-consistency test derived from the multistatic velocity model suppresses hypotheses that cannot be explained by a single target velocity, and the noise-normalized responses are non-coherently integrated for detection, localization, and velocity estimation. Compared with the CD-CZT method, the proposed UKV–IRSA avoids both the high-dimensional DOA-rate search and the Doppler-axis rescaling, thereby lowering the computational load and the sensitivity to DOA-rate errors.

The remainder of this paper is organized as follows. Section 2 introduces the system geometry and deduces the signal model of the PMR system. The proposed UKV–IRSA algorithm is described in detail in Section 3. Subsequently, Section 4 presents the simulation and experimental results to demonstrate the effectiveness of the proposed technique. Section 5 discusses the results, the modeling assumptions and the limitations of the present study. Finally, conclusions are drawn in Section 6.

## 2. System Geometry and Signal Model

**Figure 1:**
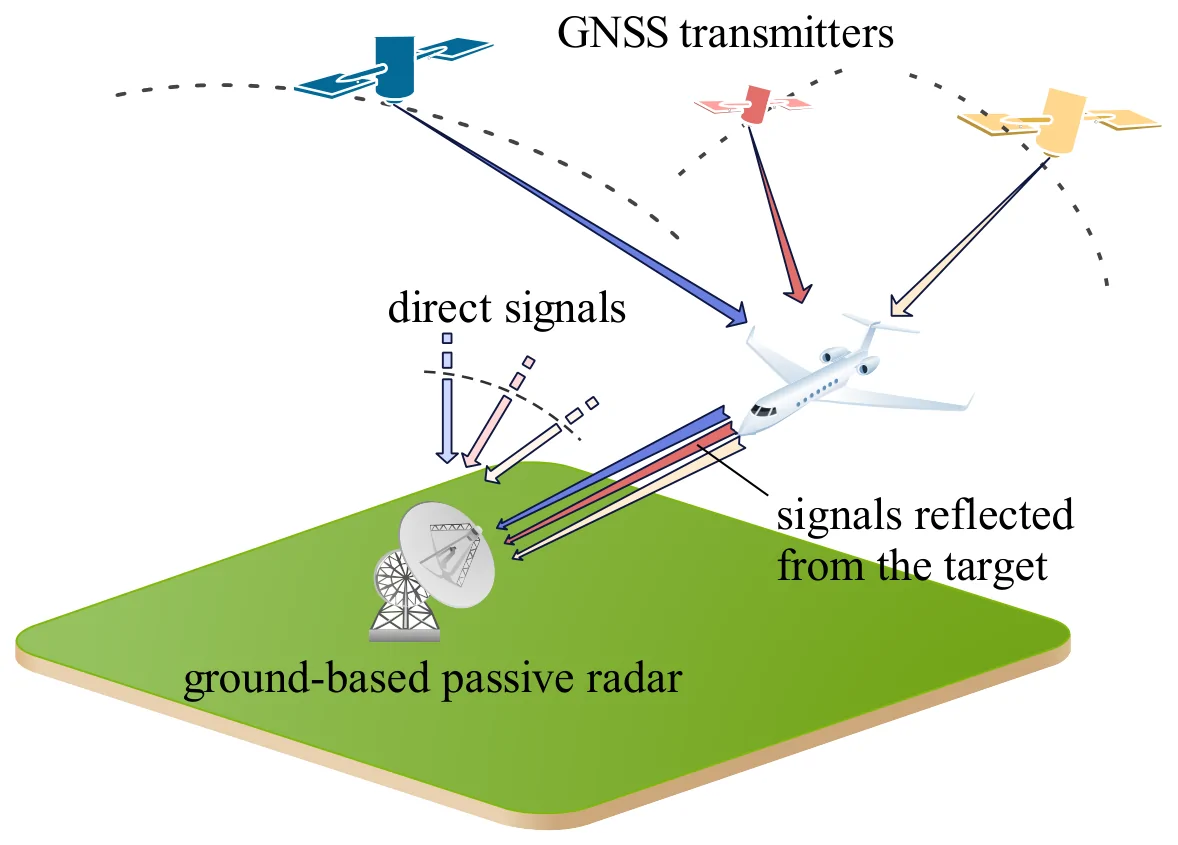
System concept for multi-GNSS transmitter passive radar for aircraft surveillance.

Figure 1 illustrates the system concept of a multi-GNSS passive radar for aircraft detection. The system comprises multiple GNSS satellites and a ground-based passive receiver, which is equipped with dual channels: a reference channel and a surveillance channel. The reference channel, which is not shown in the figure as it falls outside the primary scope of this study, employs an omnidirectional antenna to acquire direct GNSS signals for subsequent signal separation and processing. Meanwhile, the surveillance channel utilizes a high-gain antenna to collect radar echoes reflected from aerial targets. The direct-path interference (DPI) in the surveillance channel is suppressed by a sidelobe canceller (SLC). Clutter and multipath are not modeled explicitly: stationary clutter lies near zero Doppler, far from the aircraft Doppler range, and can be suppressed by moving target indication (MTI), whereas the ground-reflected echo of an aircraft is delayed by much less than one range cell relative to the direct echo and therefore only modulates the echo amplitude [33].

For a multi-illuminator passive system, three alignment steps are required to achieve time-frequency synchronization. First, the reference and surveillance channels share a local oscillator and sampling clock, defining a common receiver-time axis. Second, for all selected satellites, both channels use the same receive-time window and local starting sample; the satellites need not transmit simultaneously. Tracking each direct signal provides a satellite-specific reference replica [34], so target delay and Doppler are measured relative to the corresponding direct path and common receiver errors cancel. Third, distinct bistatic geometries yield different target delays and Doppler centroids, and it is the main content of this paper’s research.

**Figure 2:**
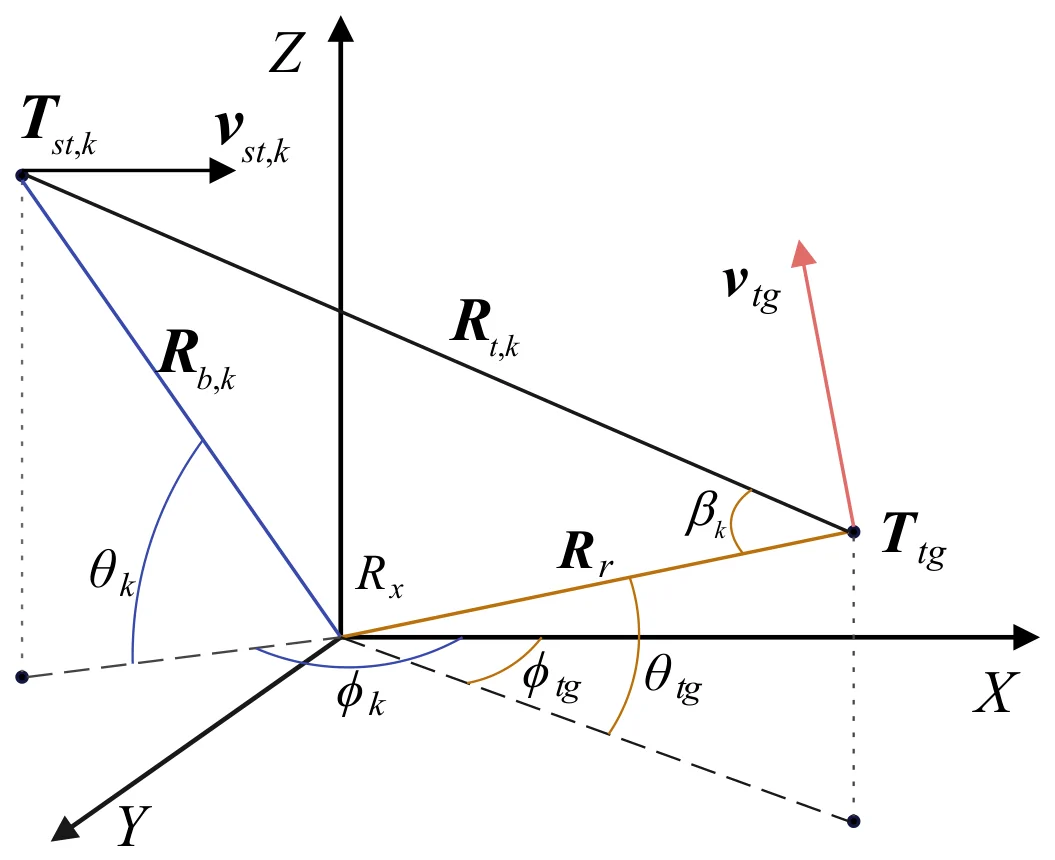
System geometry for multi-GNSS transmitter passive radar.

Figure 2 establishes a Cartesian reference frame (O,X,Y,Z) of a passive radar system, and without loss of generality, the stationary radar receiver $R_{x}$ is set as the origin *O*, and the horizontal direction of the radar line-of-sight (LOS) is defined as the *X*-axis. The reference system focuses on the *K* GNSS satellites available for detection, with the coordinates of the k-th$\left(k=1,2,\ldots ,K\right)$ transmitter and target denoted as $T_{st,k}={\left[x_{k},y_{k},z_{k}\right]}^{T}$ and $T_{tg}={\left[x,y,z\right]}^{T}$ respectively. The corresponding velocities are defined as $v_{st,k}={\left[v_{x,k},v_{y,k},v_{z,k}\right]}^{T}$, $v_{tg}={\left[v_{x},v_{y},v_{z}\right]}^{T}$. Additionally, the coordinates of the system can be re-expressed with angular relations as follows.

(1) $$\begin{array}{cc} \; & T_{st,k}=\left[\begin{array}{c} |R_{b,k}|\cos\left(\theta_{k}\right)\cos\left(\phi_{k}\right)\\ |R_{b,k}|\cos\left(\theta_{k}\right)\sin\left(\phi_{k}\right)\\ |R_{b,k}|\sin\left(\theta_{k}\right) \end{array}\right] \end{array}$$

(2) $$\begin{array}{cc} \; & T_{tg}=\left[\begin{array}{c} |R_{r}|\cos\left(\theta_{tg}\right)\cos\left(\phi_{tg}\right)\\ |R_{r}|\cos\left(\theta_{tg}\right)\sin\left(\phi_{tg}\right)\\ |R_{r}|\sin\left(\theta_{tg}\right) \end{array}\right] \end{array}$$

where $R_{b,k}=T_{st,k}-R_{x}$ is the receiver-to-satellite baseline vector, $R_{r}=T_{tg}-R_{x}$ is the receiver-to-target range vector, and $R_{t,k}=T_{st,k}-T_{tg}$ is the target-to-satellite range vector. $\left|\cdot \right|$ represents the Euclidean norm. $\phi_{k}$, $\theta_{k}$ and $\phi_{tg}$, $\theta_{tg}$ are the azimuth and elevation angles of the satellite and target, respectively.

As both satellites and targets are in constant motion, the vectors $R_{b,k}$, $R_{r}$ and $R_{t,k}$ can be written as a time-varying function with respect to satellite and target speed

(3) $$\begin{array}{cc} \; & R_{t,k}=R_{t,k}^{0}+\left(v_{st,k}-v_{tg}\right)t_{m} \end{array}$$

(4) $$\begin{array}{cc} \; & R_{r}=R_{r}^{0}+v_{tg}t_{m} \end{array}$$

(5) $$\begin{array}{cc} \; & R_{b,k}=R_{b,k}^{0}+v_{st,k}t_{m} \end{array}$$

where $R_{t,k}^{0}$, $R_{r}^{0}$ and $R_{b,k}^{0}$ are the reference range vectors at the aperture midpoint, and $t_{m}$ is the slow time measured from the aperture midpoint, with $t_{m}\in \left[-T_{a}/2,T_{a}/2\right]$ over the observation time $T_{a}$.

After both reference and surveillance channels synchronously collect signals within the observation time $T_{a}$, the reference signals from the *K* selected satellites are separated with a software GNSS receiver [34]. Artificial segmentation is required to achieve equivalent pulse signal formatting, as navigation signals are generally continuous. The equivalent pulse repetition interval (PRI) is generally set as the period of the pseudorandom noise (PRN), e.g., 1 ms for the GPS signal. After segmentation, pulse compression (PC) can be achieved through cross-correlation between the k-th reference signal and the target signal, a 2D scheme (fast-time $t\in \left[-\frac{T_{p}}{2},\frac{T_{p}}{2}\right]$ ($T_{p}$ the equivalent pulse duration) and slow-time $t_{m}\in \left[-\frac{T_{a}}{2},\frac{T_{a}}{2}\right]$) of the target signal can be expressed as

(6) $$\begin{array}{cc} S_{\mathrm{pc}}^{k}\left(t,t_{m}\right)=A_{k}\left(t_{m}\right)\exp & \left(-j2\pi \frac{R_{k}\left(t_{m}\right)}{\lambda }\right)\\ \; & \Lambda \left(t-\frac{R_{k}\left(t_{m}\right)}{c}\right)\mathrm{rect}\left(\frac{t_{m}}{T_{a}}\right) \end{array}$$

where $A_{k}$ is the complex amplitude after compression. λ is the wavelength, *c* is the speed of light. $R_{k}$ is the bistatic range of the k-th link, given by the difference between the reflected propagation path and the baseline. $\Lambda \left(\cdot \right)$ is the cross-correlation function between the reference and target signals. The resulting signal contains noise components that are typically modeled as additive white Gaussian noise. However, in practical GNSS-based passive radar scenarios, the noise may exhibit more complex characteristics, including correlated components. Fast iterative denoising methods, such as the Back to Basics (BTB) algorithm [35], offer efficient noise reduction without requiring explicit knowledge of the noise model or level, which could be incorporated as a preprocessing step to enhance the SNR before coherent integration. Since all exploited links share the GPS L1 carrier, a common wavelength λ=0.19m applies and is known at the receiver, and though $\Lambda \left(\cdot \right)$ varies with the PRN code, it can be approximated by the same triangular function.

(7) $$R_{k}=|R_{t,k}\left|+\right|R_{r}\left|-\right|R_{b,k}|$$

As [36] has proved, a square term of time is the highest order term of the range for PBR detection. Therefore, substituting (3) into (7), a second-order expansion of the bistatic range can be expressed as

(8) $$\begin{array}{cc} R_{k} & \approx R_{k}^{0}+\left[e_{t,k}^{T}v_{st,k}+{\left(e_{r}-e_{t,k}\right)}^{T}v_{tg}-e_{b,k}^{T}v_{st,k}\right]t_{m}\\ \; & +\left[\frac{|v_{st,k}-v_{tg}{|}^{2}-{\left|e_{t,k}^{T}\left(v_{st,k}-v_{tg}\right)\right|}^{2}}{2|R_{t,k}^{0}|}\;\right.\\ \; & \left.+\;\frac{|v_{tg}{|}^{2}-{\left|e_{r}^{T}v_{tg}\right|}^{2}}{2|R_{r}^{0}|}-\frac{|v_{st,k}{|}^{2}-{\left|e_{b,k}^{T}v_{st,k}\right|}^{2}}{2|R_{b,k}^{0}|}\right]t_{m}^{2} \end{array}$$

where $R_{k}^{0}=|R_{t,k}^{0}\left|+\right|R_{r}^{0}\left|-\right|R_{b,k}^{0}|$ is the initial bistatic range. et,k=Rt,k0|Rt,k0|, er=Rr0|Rr0|, and eb,k=Rb,k0|Rb,k0| are unit vectors. Let $e_{r,k}$ be the unit vector of the bistatic-range gradient with respect to the bistatic angle $\beta_{k}=∠T_{st,k}T_{tg}R_{x}$, which can be expressed as

(9) $$\begin{array}{cc} e_{r,k} & =\frac{e_{r}-e_{t,k}}{\sqrt{2+2\cos(\beta_{k})}}\\ \; & =\frac{e_{r}-e_{t,k}}{2\cos(\frac{\beta_{k}}{2})} \end{array}$$

Substituting (9) into (8), and noting that for an aircraft target close to the receiver the satellite-to-target and receiver-to-satellite curvature terms in (8) nearly cancel (since $e_{t,k}\approx e_{b,k}$, $|R_{t,k}\left|\approx \right|R_{b,k}\left|\gg \right|R_{r}|$, and $|v_{st,k}\left|\gg \right|v_{tg}|$), leaving the dominant monostatic curvature term, the bistatic range can be approximately rewritten as

(10) $$\begin{array}{cc} R_{k}\approx R_{k}^{0} & +\left[{\left(e_{t,k}-e_{b,k}\right)}^{T}v_{st,k}+2\cos\left(\frac{\beta_{k}}{2}\right)e_{r,k}^{T}v_{tg}\right]t_{m}\\ \; & +\frac{|v_{⊥,r}{|}^{2}}{2R_{r}}t_{m}^{2} \end{array}$$

where $v_{⊥,r}=v_{tg}-\left(e_{r}^{T}v_{tg}\right)e_{r}$ represents the target velocity component perpendicular to $R_{r}$. $R_{r}$ is the scalar form of $R_{r}$. From (10), the Doppler centroid and the Doppler frequency rate can be denoted as

(11) $$\left\{\begin{array}{cc} f_{\mathrm{dc},k} & =-\frac{1}{\lambda }\left[{\left(e_{t,k}-e_{b,k}\right)}^{T}v_{st,k}+2\cos\left(\frac{\beta_{k}}{2}\right)e_{r,k}^{T}v_{tg}\right]\\ f_{\mathrm{dr},k} & =-\frac{1}{\lambda }\frac{|v_{⊥,r}{|}^{2}}{R_{r}} \end{array}\right.$$

From (11), the dominant Doppler frequency rate is mainly governed by $v_{⊥,r}$ and the monostatic range $R_{r}$. Therefore, a common DFR component can be used for the selected bistatic links after the GNSS far-field approximation. The exact second-order term in (8) still contains weak satellite-dependent residuals, but these residuals are much smaller than the dominant monostatic curvature term in the considered aircraft surveillance geometry.

### Problem Analysis

**Figure 3:**
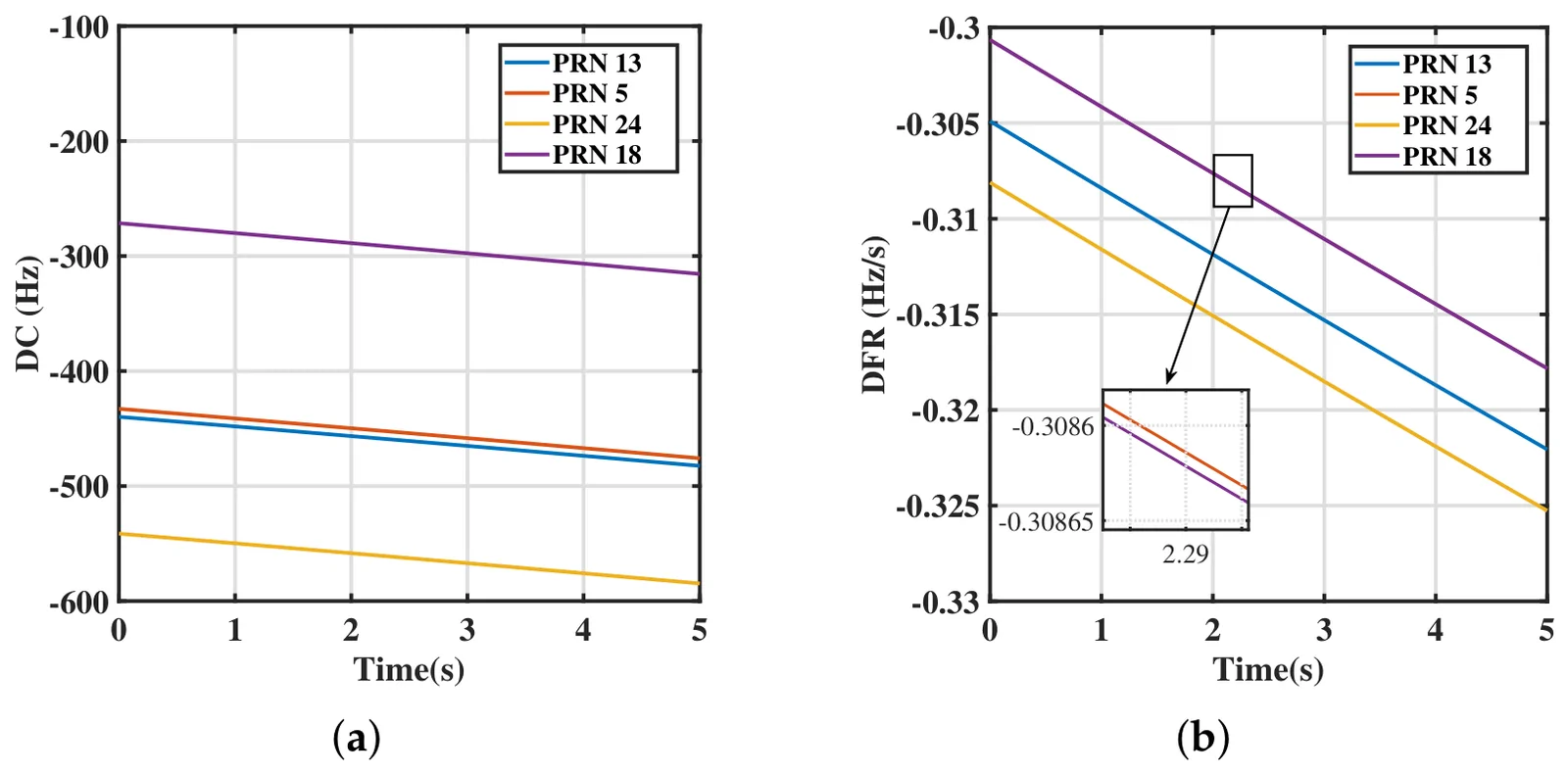
Characteristics of Doppler centroid and Doppler frequency rate from target signal (after PC). (**a**) Doppler centroid. (**b**) Doppler frequency rate.

The features of $f_{\mathrm{dc},k}$ and $f_{\mathrm{dr},k}$ in realistic geometric configurations are first analyzed through a simulation including four GPS satellites and a passive radar receiver. To conform to the parameter characteristics in the real scene, the coordinates and the velocity of satellites are received from the received GPS ephemeris information, and the receiver is geolocated at XIDIAN University. Then, an aircraft is placed at a receiver-centered relative position of approximately (31.6,5.3,2.6) km, corresponding to a slant range of about 32.1 km, with a speed of about 99 m/s. As shown in Figure 3a, the DC of echoes from all satellites exhibit distinct values, indicating coupling with its bistatic link, as deduced in (11).

Moreover, Figure 3b reveals that the DFR under different satellite illuminations is dominated by a common trend but still exhibits weak inter-link residuals and temporal variation:

1. DFR inter-link residual: the residual is caused by the nonideal cancellation between the satellite-to-target curvature and the receiver-to-satellite baseline curvature in (8). Given that the coverage range considered for GNSS-based PMR is on the order of several tens of kilometers, the corresponding DFR difference is less than 0.01 Hz/s and can be handled as a mismatch loss under the common DFR hypothesis. Quantitatively, a 0.01 Hz/s inter-link DFR difference introduces a residual quadratic phase of only $\pi \;\Delta f_{\mathrm{dr}}\;{\left(T_{n}/2\right)}^{2}\approx 0.002$ rad over a 0.5 s coherent frame, i.e., an integration loss below 0.1 dB; furthermore, this difference is finer than the DFR search grid, so the common-DFR search inherently absorbs it.
2. DFR temporal variation: as deduced in (11), DFR demonstrates explicit dependence on the monostatic range $R_{r}$. For airborne moving targets, the echo DFR demonstrates measurable fluctuations due to the changing geometrical relationship between radar and target. Through theoretical derivation, the DFR variation typically remains below 0.02 Hz/s.

These features necessitate that conventional DFR measurement-based methods employ coherent integration times exceeding one minute to achieve 0.01 Hz/s DFR resolution. At this resolution level, both inter-link residuals and temporal variations of the DFR are non-negligible, thereby rendering these approaches ineffective.

Moreover, it is impractical to achieve this Doppler resolution given the decorrelation interval of aircraft Δt, which is represented as

(12) $$\Delta t=\frac{2R\Delta \psi }{v_{t}}$$

where *R* is the target slant range and $v_{t}$ is the tangential velocity of the target relative to the receiver. Δψ is the half-width of the aspect-angle interval, measured with respect to the radar line of sight, over which the target echo remains correlated, and the factor of two corresponds to the symmetric peak-to-peak aspect span 2Δψ swept by the target. Since the LOS angular rate is $\omega =v_{t}/R$, the decorrelation interval follows as $\Delta t=2\Delta \psi /\omega =2R\Delta \psi /v_{t}$. The correlated aspect span is estimated from the two-way phase-coherence criterion Δψ≈λ/(2L), where *L* is the effective cross-range extent of the target. This criterion corresponds to the aspect rotation at which the differential two-way phase across the cross-range extent of the target becomes appreciable, so that the coherent superposition of its scatterers—and hence the echo amplitude—decorrelates [31]. For a representative aircraft with L≈60 m at λ=0.19 m, a slant range R≈32 km, and a tangential velocity $v_{t}\leq 200$ m/s, this yields Δψ≈1.6 mrad and a decorrelation interval of about 0.5 s, which is far shorter than the coherent integration time required for high-resolution DFR measurement.

Finally, these methods are designed for maritime target detection, which neglects object altitude information, confining them to 2D rather than enabling 3D localization of aerial targets.

## 3. Proposed UKV–IRSA Method

To address the above problems and achieve aircraft fusion detection, a multi-GNSS interlink data fusion method, termed UKV–IRSA, is proposed. In this method, the UKV is constructed before multistatic integration, and the link-dependent bistatic range trajectories are determined by the common DOA and monostatic range over the monostatic-range axis. Then, each bistatic link is coherently integrated along the geometry-constrained trajectory, aligned to the MRD domain through the IRSA, and fused under a Doppler-consistency constraint. In this way, target energy from different GNSS transmitters can be accumulated in the same monostatic range and zero-frequency cell without requiring DOA-rate estimation.

### 3.1. Signal Representation and DFR Hypothesis Compensation

Doppler and range migration caused by target motion hinder coherent energy accumulation within each bistatic link. To address this impairment, Doppler frequency-rate compensation (DFRC) is applied under each DFR hypothesis, and the residual DC is subsequently estimated through coherent integration along the trajectory for each link.

The received data over an aperture time $T_{a}$ are divided into $N=T_{a}/T_{n}$ frames, where the frame length $T_{n}$ is set according to the maximum coherent duration allowed by the target RCS decorrelation. For each frame, the received signal is artificially segmented into pulse signals with equivalent pulse repetition time $T_{r}$, which is equal to the repetition period of the GPS signal. All exploited transmitters share the GPS L1 carrier, so a common wavelength λ applies to every bistatic link. The local slow time in the n-th frame is defined as $\tau_{m}=mT_{r}-T_{n}/2$ with m=0,1,…,M−1 and $M=T_{n}/T_{r}$, and the corresponding absolute slow time is $t_{m}=\left(n-\frac{N-1}{2}\right)T_{n}+\tau_{m}$. After pulse compression, the target signal of the k-th bistatic link and n-th frame $\left(n=0,1,\ldots ,N-1\right)$ is

(13) $$\begin{array}{cc} S_{\mathrm{pc},n}^{k}\left(t,\tau_{m}\right)= & A_{n,k}\left(\tau_{m}\right)\mathrm{rect}\left(\frac{\tau_{m}}{T_{n}}\right)\\ \; & \times \Lambda \left(t-\frac{R_{n,k}\left(\tau_{m}\right)}{c}\right)\\ \; & \times \exp\left(-j2\pi \frac{R_{n,k}\left(\tau_{m}\right)}{\lambda }\right), \end{array}$$

where $\Lambda \left(\cdot \right)$ is the cross-correlation function between reference and target signal. Within one frame, the second-order range model is written as

(14) $$R_{n,k}\left(\tau_{m}\right)\approx R_{0,n,k}-\lambda f_{\mathrm{dc},n,k}\tau_{m}-\frac{\lambda }{2}f_{\mathrm{dr},n}{\tau_{m}}^{2}.$$

Then, the signal in (13) can be transformed into the fast-frequency and slow-time domain by the fast Fourier transform (FFT) along the fast-time axis as

(15) $$\begin{array}{cc} S_{\mathrm{pc},n}^{k}\left(f_{r},\tau_{m}\right)\; & =A_{n,k}\left(\tau_{m}\right)F\left(f_{r}\right)\mathrm{rect}\left(\frac{\tau_{m}}{T_{n}}\right)\\ \; & \times \exp\left[-j\frac{2\pi }{\lambda }R_{n,k}\left(\tau_{m}\right)\right]\\ \; & \times \exp\left[-j\frac{2\pi f_{r}}{c}R_{n,k}\left(\tau_{m}\right)\right], \end{array}$$

where F(·) denotes the Fourier transform of Λ(·), and $f_{r}$ is the fast-time (range) frequency. Substituting the intra-frame range model (14) into (15) yields

(16) $$\begin{array}{cc} \; & S_{\mathrm{pc},n}^{k}\left(f_{r},\tau_{m}\right)=A_{n,k}\left(\tau_{m}\right)F\left(f_{r}\right)\mathrm{rect}\left(\frac{\tau_{m}}{T_{n}}\right)\\ \; & \times \exp\left[-j2\pi \left(\frac{R_{0,n,k}}{\lambda }-f_{\mathrm{dc},n,k}\tau_{m}-\frac{f_{\mathrm{dr},n}}{2}{\tau_{m}}^{2}\right)\right]\\ \; & \times \exp\left[-j\frac{2\pi f_{r}}{c}\left(R_{0,n,k}-\lambda f_{\mathrm{dc},n,k}\tau_{m}-\frac{\lambda }{2}f_{\mathrm{dr},n}{\tau_{m}}^{2}\right)\right]. \end{array}$$

**Figure 4:**
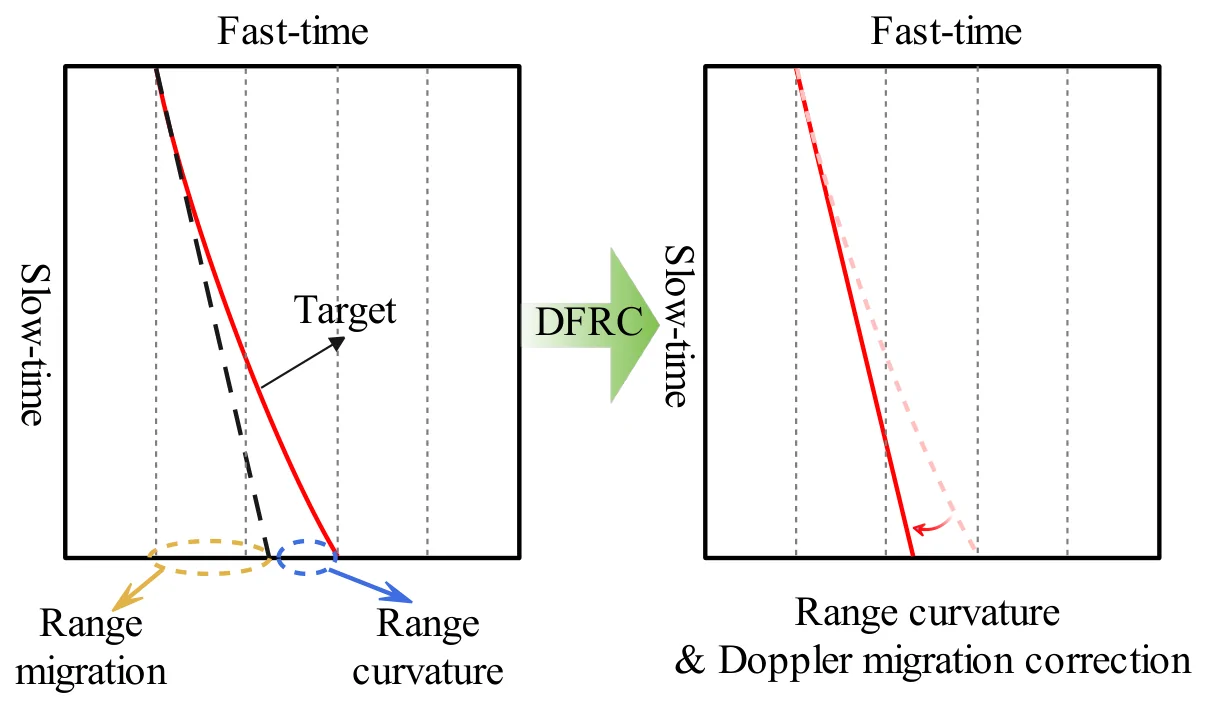
DFRC processing.

The first exponential term in (16) is the carrier phase, whereas the second exponential term is the fast-frequency phase caused by the target delay. The coupling terms involving $f_{r}\tau_{m}$ and $f_{r}{\tau_{m}}^{2}$ introduce range migration and range curvature effects, respectively. As analyzed in Section 2, the DFR is governed by a common term whose inter-link variation is negligible—finer than the DFR search grid and corresponding to an integration loss below 0.1 dB over a coherent frame. The DFR can therefore be treated as approximately link-invariant and is denoted by $f_{\mathrm{dr},n}$ hereafter. The proposed method does not require an externally measured high-resolution DFR; instead, within each short coherent frame the DFR is treated as a bounded hypothesis $f_{\mathrm{dr}}$ and searched. Accordingly, a bounded DFR search set is introduced as

(17) $$F_{\mathrm{dr}}=\left\{f_{\mathrm{dr},l}=f_{\mathrm{dr},\min}+l\Delta f_{\mathrm{dr}},\;l=0,1,\ldots ,N_{\mathrm{dr}}-1\right\},$$

where $\Delta f_{\mathrm{dr}}=\left(f_{\mathrm{dr},\max}-f_{\mathrm{dr},\min}\right)/\left(N_{\mathrm{dr}}-1\right)$, and $f_{\mathrm{dr},\min}$ and $f_{\mathrm{dr},\max}$ are determined by the expected target velocity and range interval. Under the sign convention in (11), a practical bound can be selected as $-V_{\max}^{2}/\left(\lambda R_{\min}\right)\leq f_{\mathrm{dr}}\leq 0$. For each candidate $f_{\mathrm{dr}}\in F_{\mathrm{dr}}$, DFRC is applied to (15) as

(18) $$\begin{array}{cc} S_{\mathrm{DFRC},n}^{k}\left(f_{r},\tau_{m};f_{\mathrm{dr}}\right) & =S_{\mathrm{pc},n}^{k}\left(f_{r},\tau_{m}\right)\\ \; & \times \exp\left(-j\pi \frac{f_{c}+f_{r}}{f_{c}}f_{\mathrm{dr}}{\tau_{m}}^{2}\right), \end{array}$$

where $f_{\mathrm{dr}}$ is the DFR hypothesis under test and $f_{c}=c/\lambda$ is the carrier frequency. The compensation term in (18) jointly removes the quadratic phase in both the carrier phase and the fast-frequency phase when $f_{\mathrm{dr}}$ matches the true DFR. After DFRC, the fast-time and slow-time domain signal can be obtained by the inverse fast Fourier transform (IFFT) in the fast-frequency domain

(19) $$\begin{array}{cc} S_{\mathrm{DFRC},n}^{k} & \left(t,\tau_{m};f_{\mathrm{dr}}\right)=A_{n,k}\left(\tau_{m}\right)\mathrm{rect}\left(\frac{\tau_{m}}{T_{n}}\right)\\ \; & \times \Lambda \left(t-\tau_{0,n,k}\left(\tau_{m}\right)\right)\\ \; & \times \exp\left[-j2\pi \left(\frac{R_{0,n,k}}{\lambda }-f_{\mathrm{dc},n,k}\tau_{m}\right)\right], \end{array}$$

where $\tau_{0,n,k}\left(\tau_{m}\right)=\frac{R_{0,n,k}-\lambda f_{\mathrm{dc},n,k}\tau_{m}}{c}$. From the result of (19), the DFR term is eliminated for the matched hypothesis ($f_{\mathrm{dr}}=f_{\mathrm{dr},n}$), whereas a residual quadratic phase remains for mismatched hypotheses and causes integration loss. The sketch of the DFRC is shown in Figure 4. The DC and DFR embedded in the echo induce range migration and range curvature of the target in the fast-slow time domain, respectively. Additionally, the DFR further contributes to Doppler walk during coherent accumulation, resulting in target energy dispersion and accumulation loss.

### 3.2. Unified Kinematic Vector and Coherent Integration

It is noticeable that the target position is coupled with the local slow time $\tau_{m}$. To correct the intra-link range migration under a multistatic constraint, the DFRC output is first transformed to the bistatic range and slow-time domain, where the fast time *t* is mapped to the range-domain sampling variable $R^{\prime }=ct$

(20) $$\begin{array}{cc} S_{\mathrm{DFRC},n}^{k} & \left(R^{\prime },\tau_{m};f_{\mathrm{dr}}\right)=A_{n,k}\left(\tau_{m}\right)\mathrm{rect}\left(\frac{\tau_{m}}{T_{n}}\right)\\ \; & \times \Lambda \left(\frac{R^{\prime }}{c}-\tau_{0,n,k}\left(\tau_{m}\right)\right)\\ \; & \times \exp\;\left[-j2\pi \;\left(\frac{R_{0,n,k}}{\lambda }-f_{\mathrm{dc},n,k}\tau_{m}\right)\right]. \end{array}$$

The bistatic range (10) is rewritten as

(21) $$R_{k}=R_{r}+\Delta R_{k},$$

where $\Delta R_{k}=|R_{t,k}\left|-\right|R_{b,k}|$ is the extra range that the k-th transmitter contributes. Taking a far-field Taylor expansion under the coordinate of the receiver $R_{x}$, (21) can be approximately rewritten as

(22) $$\Delta R_{k}\approx -\frac{T_{tg}^{T}T_{st,k}}{|R_{b,k}|}=-\frac{xx_{k}+yy_{k}+zz_{k}}{\sqrt{x_{k}^{2}+y_{k}^{2}+z_{k}^{2}}}.$$

According to the space geometry relation from (1) and (2), we obtain

(23) $$\begin{array}{cc} \Delta R_{k}\approx & -R_{r}\cos\theta_{k}\left[\cos\left(\phi_{k}-\phi_{tg}\right)\cos\theta_{tg}\right]\\ \; & -R_{r}\sin\theta_{k}\sin\theta_{tg}. \end{array}$$

Hence, the bistatic range can be factorized into a common monostatic range and a link-dependent geometric scaling factor by substituting (23) into (21)

(24) $$\begin{array}{cc} R_{k} & =R_{r}\left\{1-\cos\theta_{k}\cos\theta_{tg}\cos\left(\phi_{k}-\phi_{tg}\right)-\sin\theta_{k}\sin\theta_{tg}\right\}\\ \; & =R_{r}\xi_{k}\left(\phi_{tg},\theta_{tg}\right), \end{array}$$

where $\xi_{k}\left(\phi_{tg},\theta_{tg}\right)$ is the scaling factor of the k-th bistatic link determined by the target DOA. $\phi_{tg}$ and $\theta_{tg}$ denote the azimuth and elevation angle hypotheses of the target DOA, respectively. It should be noted that the target DOA can be searched within the beamwidth of the beam-pointing angle of the radar receiver, and the search range is as follows

(25) $$\begin{array}{cc} \phi_{tg} & =\phi_{0}-\phi_{3\mathrm{dB}}:\delta_{\phi }:\phi_{0}+\phi_{3\mathrm{dB}} \end{array}$$

(26) $$\begin{array}{cc} \theta_{tg} & =\theta_{0}-\theta_{3\mathrm{dB}}:\delta_{\theta }:\theta_{0}+\theta_{3\mathrm{dB}} \end{array}$$

where $\phi_{0}$ and $\theta_{0}$ denote the azimuth angle and elevation angle of the radar beam pointing, respectively. $\phi_{3\mathrm{dB}}$ and $\theta_{3\mathrm{dB}}$ are the radar azimuth and elevation beamwidths, respectively. $\delta_{\phi }$ and $\delta_{\theta }$ represent the target azimuth and elevation search intervals, respectively.

The proposed UKV is defined as

(27) $$\chi =\left(\phi_{tg},\theta_{tg},R_{r}^{\prime }\right)\in \Omega_{\phi }\times \Omega_{\theta }\times \Omega_{R},$$

which comprises only the parameters that are invariant across all bistatic links, since every transmitter–receiver pair observes the same target. The bistatic range and the Doppler centroid, by contrast, are link-dependent. Here $R_{r}^{\prime }\in \Omega_{R}$ is the common monostatic-range hypothesis used for interlink registration; it is the sampled counterpart of the physical receiver-to-target range $R_{r}=\left|R_{r}\right|$, whereas $R_{k}^{\prime }$ denotes the processed-domain bistatic-range axis of the k-th link. The DFR hypothesis $f_{\mathrm{dr}}\in F_{\mathrm{dr}}$ remains an independent search variable. For a given χ, the initial bistatic range of the k-th link is determined by

(28) $$R_{0,k}^{\prime }=\xi_{k}\left(\phi_{tg},\theta_{tg}\right)R_{r}^{\prime }.$$

Because the scaling factor $\xi_{k}$ is governed only by the link-invariant DOA, which is approximately constant over the short multi-frame aperture, the frame index *n* is omitted from $R_{0,k}^{\prime }$; the frame-dependent range walk of the target is instead compensated separately by the inter-frame registration in (37). Thus, the RFT trajectory used by each bistatic link is generated by the UKV as

(29) $$R_{k}^{\prime }\left(\tau_{m};\chi ,f^{\prime }\right)=R_{0,k}^{\prime }-\lambda f^{\prime }\tau_{m}.$$

For the airplane of interest with maximum velocity $V_{\max}$, the Doppler search set is denoted by F, with $-2V_{\max}/\lambda \leq f^{\prime }\leq 2V_{\max}/\lambda$. The coherent integration output is written as

(30) $$\begin{array}{cc} H_{n,k}\left(\chi ,f_{\mathrm{dr}},f^{\prime }\right)=\sum_{\tau_{m}} & S_{\mathrm{DFRC},n}^{k}\left(R_{k}^{\prime }\left(\tau_{m};\chi ,f^{\prime }\right),\tau_{m};f_{\mathrm{dr}}\right)\\ \; & \times \exp(-j2\pi f^{\prime }\tau_{m}). \end{array}$$

The frequency corresponding to the peak of (30) represents the DC most likely carried by the target signal under the joint hypotheses χ and $f_{\mathrm{dr}}$, which is expressed as

(31) $${\tilde{f}}_{\mathrm{dc},n,k}\left(\chi ,f_{\mathrm{dr}}\right)=\underset{f^{\prime }\in F}{\arg\;\max}{\left|H_{n,k}\left(\chi ,f_{\mathrm{dr}},f^{\prime }\right)\right|}^{2}.$$

The corresponding integration statistic is denoted by

(32) $$p_{n,k}\left(\chi ,f_{\mathrm{dr}}\right)=\max_{f^{\prime }\in F}{\left|H_{n,k}\left(\chi ,f_{\mathrm{dr}},f^{\prime }\right)\right|}^{2},$$

where ${\tilde{f}}_{\mathrm{dc},n,k}\left(\chi ,f_{\mathrm{dr}}\right)$ is the Doppler frequency at which the peak occurs, while $p_{n,k}\left(\chi ,f_{\mathrm{dr}}\right)$ is the peak power used as the integration statistic.

**Figure 5:**
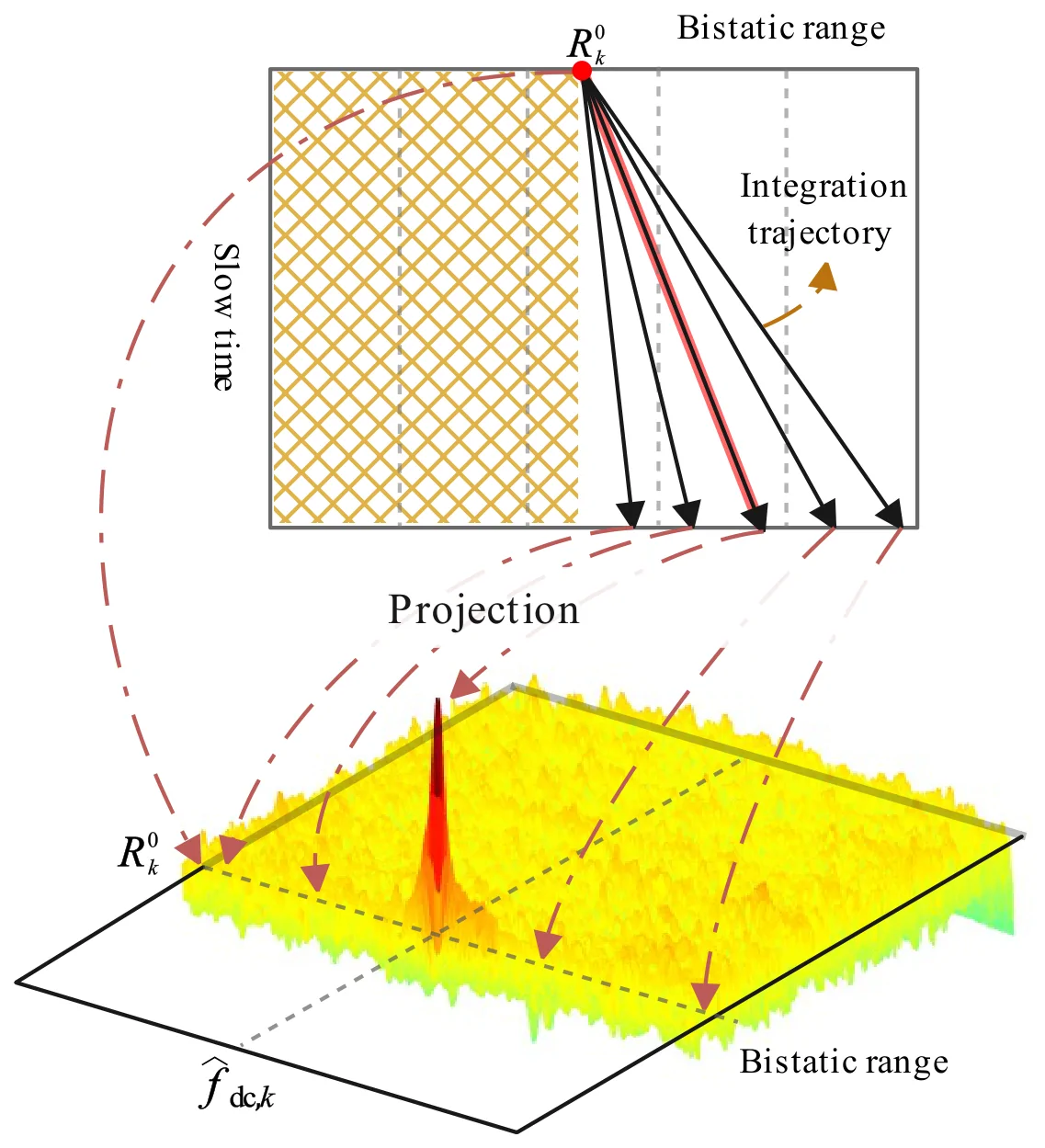
DC estimation processing under the UKV.

The sketch of the DC estimation is shown in Figure 5. Different from conventional single-link RFT processing, the initial range $R_{0,k}^{\prime }$ in (29) is not independently searched for each bistatic link. It is registered from the same DOA hypothesis along the common monostatic-range axis through (28), while the quadratic phase is selected from the common DFR hypothesis in (17). Therefore, the proposed coherent integration is a geometry-constrained multistatic search rather than repeated independent RFT processing. A target peak can be reinforced only when the DFR hypothesis, the DOA hypothesis, and the link-dependent DC estimates are mutually consistent.

This division of roles underlies the robustness of the proposed search. The initial range $R_{0,k}^{\prime }=\xi_{k}\left(\phi_{tg},\theta_{tg}\right)R_{r}^{\prime }$ in (28) couples the DOA to the long monostatic range $R_{r}^{\prime }$, so a DOA error is scaled by $R_{r}^{\prime }$. This product, however, only registers the target on the range axis: it perturbs the zeroth-order range offset, whose tolerance is one resolution cell and which does not accumulate over the integration time, so a DOA error broadens the cross-link registration gracefully rather than destroying coherence. By contrast, coherent integration is governed by the slow-time quadratic phase, and keeping its residual within a fraction of a cycle over the aperture bounds the admissible chirp-coefficient (range-curvature) error to the order of $\lambda /T_{n}^{2}$, a tolerance tightened by the long aperture $T_{n}$. Under the conventional angle-rate parameterization, this quadratic phase is produced by the transverse motion in proportion to the range and the squared angle rate, $∼R_{r}{\dot{\theta }}^{2}$, so an angle-rate error maps to a chirp error of $∼R_{r}\dot{\theta }\;\delta \dot{\theta }$, scaled by the same long range and then magnified over $T_{n}^{2}$. The proposed processing instead handles the quadratic phase through the independent bounded DFR hypothesis $f_{\mathrm{dr}}$, whose mapping to the chirp coefficient is a range-independent constant proportional to λ. The range leverage is thereby confined to the tolerant registration term and removed from the migration term that limits coherence, which is the underlying reason why the bounded DFR search is far less sensitive to estimation errors than the conventional DOA-rate search.

Then, DC compensation is applied along the hypothesis-driven target trajectory. Consequently, the coherently integrated BRD response of the k-th bistatic link is obtained as

(33) $$\begin{array}{cc} \; & G_{n}^{k}\left(R_{k}^{\prime },f;\chi ,f_{\mathrm{dr}}\right)\\ \; & =\sum_{\tau_{m}}S_{\mathrm{DFRC},n}^{k}\left(R_{k}^{\prime }-\lambda {\tilde{f}}_{\mathrm{dc},n,k}\left(\chi ,f_{\mathrm{dr}}\right)\tau_{m},\tau_{m};f_{\mathrm{dr}}\right)\\ \; & \;\times \exp\left[-j2\pi {\tilde{f}}_{\mathrm{dc},n,k}\left(\chi ,f_{\mathrm{dr}}\right)\tau_{m}\right]\exp\left(-j2\pi f\tau_{m}\right). \end{array}$$

The arguments $\left(\chi ,f_{\mathrm{dr}}\right)$ indicate that the BRD response is formed under the current UKV and DFR hypotheses through the link-dependent DC estimate ${\tilde{f}}_{\mathrm{dc},n,k}\left(\chi ,f_{\mathrm{dr}}\right)$, even though the integration itself is sampled along the bistatic-range axis $R_{k}^{\prime }$. When both hypotheses are matched, the target power is accumulated at the link-dependent bistatic range $R_{0,k}^{\prime }$ and zero Doppler. However, this integrated position is still expressed in the bistatic range coordinate and must be registered before non-coherent fusion.

### 3.3. Interlink Range Scaling Alignment and Doppler-Consistent Fusion

Substituting (24) into (33), a MRD response can be obtained with the target located at the same monostatic-range position. Since $R_{k}^{\prime }=\xi_{k}\left(\phi_{tg},\theta_{tg}\right)R_{r}^{\prime }$, evaluating the monostatic range grid $R_{r}^{\prime }$ requires scaled sampling of the k-th BRD map at the corresponding bistatic range $R_{k}^{\prime }$. The IRSA is implemented by a one-dimensional CZT operator,

(34) $$\begin{array}{cc} G_{n,k}^{\mathrm{MRD}}\left(f;\chi ,f_{\mathrm{dr}}\right)\; & =C_{\xi_{k}}^{R}\left\{G_{n}^{k}\left(R_{k}^{\prime },f;\chi ,f_{\mathrm{dr}}\right)\right\}\left(R_{r}^{\prime }\right)\\ \; & =G_{n}^{k}\;\left(\xi_{k}R_{r}^{\prime },f;\chi ,f_{\mathrm{dr}}\right), \end{array}$$

where $C_{\xi_{k}}^{R}\left\{\cdot \right\}$ denotes the CZT-based range scaling with ratio $\xi_{k}$ that realizes the IRSA. Compared with CD-CZT, only the range axis is resampled in (34); Doppler-axis rescaling and DOA-rate search are not required because the link-dependent DC has been estimated and compensated during coherent integration.

For a candidate UKV, the DC measurements from different links should be generated by the same target velocity. The frame-averaged DC of the k-th link is calculated as

(35) $${\bar{f}}_{\mathrm{dc},k}\left(\chi ,f_{\mathrm{dr}}\right)=\frac{\sum_{n=0}^{N-1}p_{n,k}\left(\chi ,f_{\mathrm{dr}}\right){\tilde{f}}_{\mathrm{dc},n,k}\left(\chi ,f_{\mathrm{dr}}\right)}{\sum_{n=0}^{N-1}p_{n,k}\left(\chi ,f_{\mathrm{dr}}\right)}.$$

Then, the Doppler-consistency residual is defined as

(36) $$\rho_{\mathrm{dc}}\left(\chi ,f_{\mathrm{dr}}\right)={∥{\bar{f}}_{\mathrm{dc}}\left(\chi ,f_{\mathrm{dr}}\right)-D{\hat{v}}_{tg}\left(\chi ,f_{\mathrm{dr}}\right)-b∥}_{2}^{2},$$

where ${\bar{f}}_{\mathrm{dc}}={\left[{\bar{f}}_{\mathrm{dc},1},{\bar{f}}_{\mathrm{dc},2},\ldots ,{\bar{f}}_{\mathrm{dc},K}\right]}^{T}$. The matrix D and vector b are constructed under the tested UKV, and ${\hat{v}}_{tg}\left(\chi ,f_{\mathrm{dr}}\right)$ is obtained from the least-squares velocity model given later in (46). Since ${\hat{v}}_{tg}$ is the least-squares solution, the Doppler-consistency residual discriminates only when the links are redundant (K>3), where the velocity system is over-determined and the residual is generally nonzero.

Because the aircraft also translates in range over the multi-frame aperture, its monostatic range drifts from frame to frame, even though every link is compensated to zero Doppler within a frame. Before non-coherent accumulation, each frame is registered to the initial frame: using the velocity ${\hat{v}}_{tg}$ estimated from the preceding frames, the range walk accumulated by the n−th frame along the receiver line of sight $e_{r}$ is $\left(e_{r}^{T}{\hat{v}}_{tg}\right)\;nT_{n}$, and the MRD response is shifted to keep the target at the initial-frame range,

(37) $$G_{n,k}^{\mathrm{MRD}}\left(f;\chi ,f_{\mathrm{dr}}\right)\;\leftarrow \;G_{n,k}^{\mathrm{MRD}}\;\left(f;\chi_{n}^{+},f_{\mathrm{dr}}\right),\;\chi_{n}^{+}=\left(\phi_{tg},\theta_{tg},R_{r}^{\prime }+\left(e_{r}^{T}{\hat{v}}_{tg}\right)\;nT_{n}\right).$$

This space-time hybrid registration confines the energy of all frames to the same monostatic range $R_{r}^{\prime }$ prior to the non-coherent fusion. The fused decision statistic is constructed as

(38) $$J\left(\chi ,f_{\mathrm{dr}}\right)=\sum_{k=1}^{K}\sum_{n=0}^{N-1}\frac{{\left|G_{n,k}^{\mathrm{MRD}}\left(0;\chi ,f_{\mathrm{dr}}\right)\right|}^{2}}{{\hat{\sigma }}_{n,k}^{2}}-\gamma_{\mathrm{dc}}\rho_{\mathrm{dc}}\left(\chi ,f_{\mathrm{dr}}\right),$$

where ${\hat{\sigma }}_{n,k}^{2}$ is the noise power estimated from guard-free range-Doppler cells, and $\gamma_{\mathrm{dc}}>0$ is a weighting factor for the Doppler-consistency penalty, with units that scale the Hz^2^ residual to the noise-normalized statistic and tuned to the desired false-alarm rate. Because the bistatic RCS varies across links with the forward-scattering gain and the target aspect, the per-link echoes generally differ in strength. As (38) performs noise-normalized non-coherent integration, such RCS disparities only reweight the individual link contributions without biasing the detector, while the spatially diverse links jointly furnish a diversity gain against target amplitude fluctuation [31,37]. The per-link RCS therefore need not be modeled explicitly in (38). The target parameters are estimated by

(39) $$\left(\hat{\chi },{\hat{f}}_{\mathrm{dr}}\right)=\underset{\begin{array}{c} \chi \in \Omega_{\phi }\times \Omega_{\theta }\times \Omega_{R}\\ f_{\mathrm{dr}}\in F_{\mathrm{dr}} \end{array}}{\arg\;\max}J\left(\chi ,f_{\mathrm{dr}}\right).$$

After $\hat{\chi }=\left({\hat{\phi }}_{tg},{\hat{\theta }}_{tg},{\hat{R}}_{r}^{\prime }\right)$ and ${\hat{f}}_{\mathrm{dr}}$ are obtained, the fused MRD map for display can be written as

(40) $$G_{r}\left(R_{r}^{\prime },f;{\hat{\phi }}_{tg},{\hat{\theta }}_{tg},{\hat{f}}_{\mathrm{dr}}\right)=\sum_{k=1}^{K}\sum_{n=0}^{N-1}\frac{{\left|G_{n,k}^{\mathrm{MRD}}\;\left(f;\left({\hat{\phi }}_{tg},{\hat{\theta }}_{tg},R_{r}^{\prime }\right),{\hat{f}}_{\mathrm{dr}}\right)\right|}^{2}}{{\hat{\sigma }}_{n,k}^{2}},$$

which displays the noise-normalized fused response over the monostatic-range axis $R_{r}^{\prime }$ and Doppler *f* at the estimated angular and DFR hypotheses.

### 3.4. Target Localization and Velocity Estimation

Based on the UKV peak obtained from (39), i.e., the monostatic range ${\hat{R}}_{r}^{\prime }$ and the DOA estimates ${\hat{\phi }}_{tg}$, ${\hat{\theta }}_{tg}$, the target position can be calculated as follows

(41) $$\begin{array}{c} {\hat{T}}_{tg}=\left[\begin{array}{c} {\hat{R}}_{r}^{\prime }\cos\left({\hat{\theta }}_{tg}\right)\cos\left({\hat{\phi }}_{tg}\right)\\ {\hat{R}}_{r}^{\prime }\cos\left({\hat{\theta }}_{tg}\right)\sin\left({\hat{\phi }}_{tg}\right)\\ {\hat{R}}_{r}^{\prime }\sin\left({\hat{\theta }}_{tg}\right). \end{array}\right] \end{array}$$

The velocity of the target can be estimated from the Doppler centroids of the selected bistatic links. For a tested UKV and DFR hypothesis, the DC measurement vector is ${\bar{f}}_{\mathrm{dc}}\left(\chi ,f_{\mathrm{dr}}\right)$. From the first-order term of (8), the bistatic range rate is

(42) $${\dot{R}}_{k}={\left(e_{t,k}-e_{b,k}\right)}^{T}v_{st,k}+{\left(e_{r}-e_{t,k}\right)}^{T}v_{tg}.$$

Since $f_{\mathrm{dc},k}=-{\dot{R}}_{k}/\lambda$, the Doppler centroid measurements can be written as a linear model

(43) $${\bar{f}}_{\mathrm{dc}}=Dv_{tg}+b+\epsilon ,$$

where ϵ denotes the Doppler centroid estimation error, and the k-th row of D and the k-th element of b are

(44) $$\begin{array}{cc} d_{k}^{T}\; & =-\frac{1}{\lambda }{\left(e_{r}-e_{t,k}\right)}^{T}, \end{array}$$

(45) $$\begin{array}{cc} b_{k}\; & =-\frac{1}{\lambda }{\left(e_{t,k}-e_{b,k}\right)}^{T}v_{st,k}. \end{array}$$

Thus, $D={\left[d_{1},d_{2},\ldots ,d_{K}\right]}^{T}$ and $b={\left[b_{1},b_{2},\ldots ,b_{K}\right]}^{T}$. The target velocity under χ and $f_{\mathrm{dr}}$ is estimated by the Moore-Penrose pseudoinverse

(46) $${\hat{v}}_{tg}\left(\chi ,f_{\mathrm{dr}}\right)=D^{†}\left({\bar{f}}_{\mathrm{dc}}-b\right).$$

When D has full column rank, (46) reduces to the least-squares solution

(47) $${\hat{v}}_{tg}\left(\chi ,f_{\mathrm{dr}}\right)={\left(D^{T}D\right)}^{-1}D^{T}\left({\bar{f}}_{\mathrm{dc}}-b\right).$$

For K>3, this expression gives the least-squares estimate; for K=3 and nonsingular geometry, it reduces to a direct solution. If D is rank deficient, the 3D velocity is not fully observable from the selected bistatic links. The final target velocity is obtained as ${\hat{v}}_{tg}={\hat{v}}_{tg}\left(\hat{\chi },{\hat{f}}_{\mathrm{dr}}\right)$.

The proposed UKV–IRSA processing is summarized in Algorithm 1.

### 3.5. Flowchart of the UKV–IRSA Procedure

**Figure 6:**
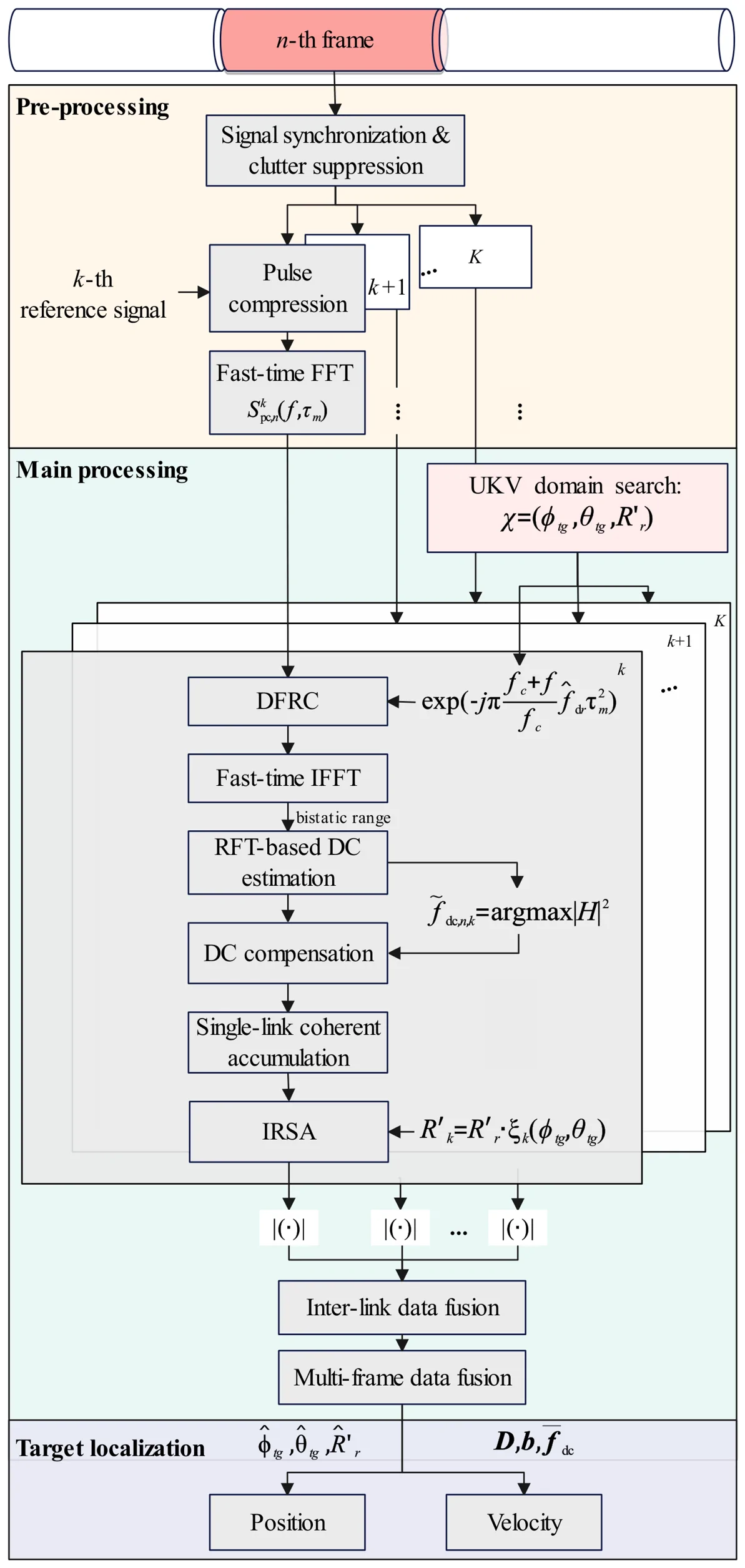
UKV–IRSA processing chain. The signal of the *n*-th frame is pulse-compressed with the *K* reference signals and fused across the links by UKV–IRSA; the results of all frames are then fused for target detection, localization and velocity estimation.

The flowchart of the proposed UKV–IRSA algorithm is presented in Figure 6. For each DFR hypothesis, DFRC is first utilized to eliminate the intra-link range curvature under the tested quadratic phase model. Then, the DOA of the UKV, together with the common monostatic-range axis, determines the bistatic RFT trajectories of all available links. After link-wise DC estimation and coherent integration, the BRD responses are mapped into the MRD domain by the IRSA. Finally, the Doppler-consistency residual and the noise-normalized non-coherent fusion statistic are jointly evaluated, and the target can be correctly detected and positioned at the peak of the fused statistic.
**Algorithm 1** Proposed UKV–IRSA method.INPUT $S_{\mathrm{pc},n}^{k}\left(f_{r},\tau_{m}\right)$, satellite geometry, $\Omega_{R}$, $\Omega_{\phi }$, $\Omega_{\theta }$, $F_{\mathrm{dr}}$, and F   $\mathrm{for}\;f_{\mathrm{dr}}\in F_{\mathrm{dr}}$       Apply DFRC using (18) and obtain $S_{\mathrm{DFRC},n}^{k}\left(R^{\prime },\tau_{m};f_{\mathrm{dr}}\right)$       $\mathrm{for}\;\chi =\left(\phi_{tg},\theta_{tg},R_{r}^{\prime }\right)\in \Omega_{\phi }\times \Omega_{\theta }\times \Omega_{R}$ **in a vectorized manner**           for k=1:K,n=0:N−1           Compute $R_{0,k}^{\prime }=\xi_{k}\left(\phi_{tg},\theta_{tg}\right)R_{r}^{\prime }$ via (28)           $\mathrm{for}\;f^{\prime }\in F$               Compute $H_{n,k}\left(\chi ,f_{\mathrm{dr}},f^{\prime }\right)$ along (29) using (30)           end           ${\tilde{f}}_{\mathrm{dc},n,k}\left(\chi ,f_{\mathrm{dr}}\right)\leftarrow {\arg\;\max}_{f^{\prime }\in F}{\left|H_{n,k}\left(\chi ,f_{\mathrm{dr}},f^{\prime }\right)\right|}^{2}$,
and $p_{n,k}\left(\chi ,f_{\mathrm{dr}}\right)\leftarrow \max_{f^{\prime }\in F}{\left|H_{n,k}\left(\chi ,f_{\mathrm{dr}},f^{\prime }\right)\right|}^{2}$           DC-compensate and integrate, then align to $G_{n,k}^{\mathrm{MRD}}\left(f;\chi ,f_{\mathrm{dr}}\right)$ by IRSA (34)       end       Compute ${\bar{f}}_{\mathrm{dc},k}\left(\chi ,f_{\mathrm{dr}}\right)$ via (35)       Construct D and b under χ; estimate ${\hat{v}}_{tg}\left(\chi ,f_{\mathrm{dr}}\right)$ via (46)       Compute $\rho_{\mathrm{dc}}\left(\chi ,f_{\mathrm{dr}}\right)$ via (36)       Register frames via (37) and compute $J(\chi ,f_{\mathrm{dr}})$ via (38)    end  end  $\left(\hat{\chi },{\hat{f}}_{\mathrm{dr}}\right)\leftarrow {\arg\;\max}_{\chi ,\;f_{\mathrm{dr}}}J\left(\chi ,f_{\mathrm{dr}}\right)$**return** $\hat{\chi }$, ${\hat{f}}_{\mathrm{dr}}$, $G_{r}\left(R_{r}^{\prime },f;{\hat{\phi }}_{tg},{\hat{\theta }}_{tg},{\hat{f}}_{\mathrm{dr}}\right)$, and ${\hat{v}}_{tg}$

### 3.6. Computational Complexity

The computational cost is evaluated in real-valued floating-point operations (FLOPs), with each complex multiplication and addition counted as six and two FLOPs, respectively. Let $N_{p}$ and $N_{t}$ denote the numbers of azimuth and elevation search points, respectively; $N_{R}$, $N_{f}$, and *M* denote the numbers of processed range cells, Doppler cells, and slow-time samples per frame; $N_{\mathrm{fr}}$ is the number of processing frames; $N_{\mathrm{dr}}$ is the number of DFR hypotheses; and *K* is the number of bistatic links. For each tested UKV, the dominant cost arises from the RFT-based coherent integration, while the IRSA introduces an additional one-dimensional CZT-based range-scaling operation. Neglecting lower-order terms, the overall complexity is

(48) $$O\left(KN_{\mathrm{fr}}N_{\mathrm{dr}}N_{p}N_{t}N_{R}\left(N_{f}M+N_{f}\log_{2}N_{R}\right)\right),$$

where the two terms in parentheses correspond to coherent integration and IRSA, respectively.

**Figure 7:**
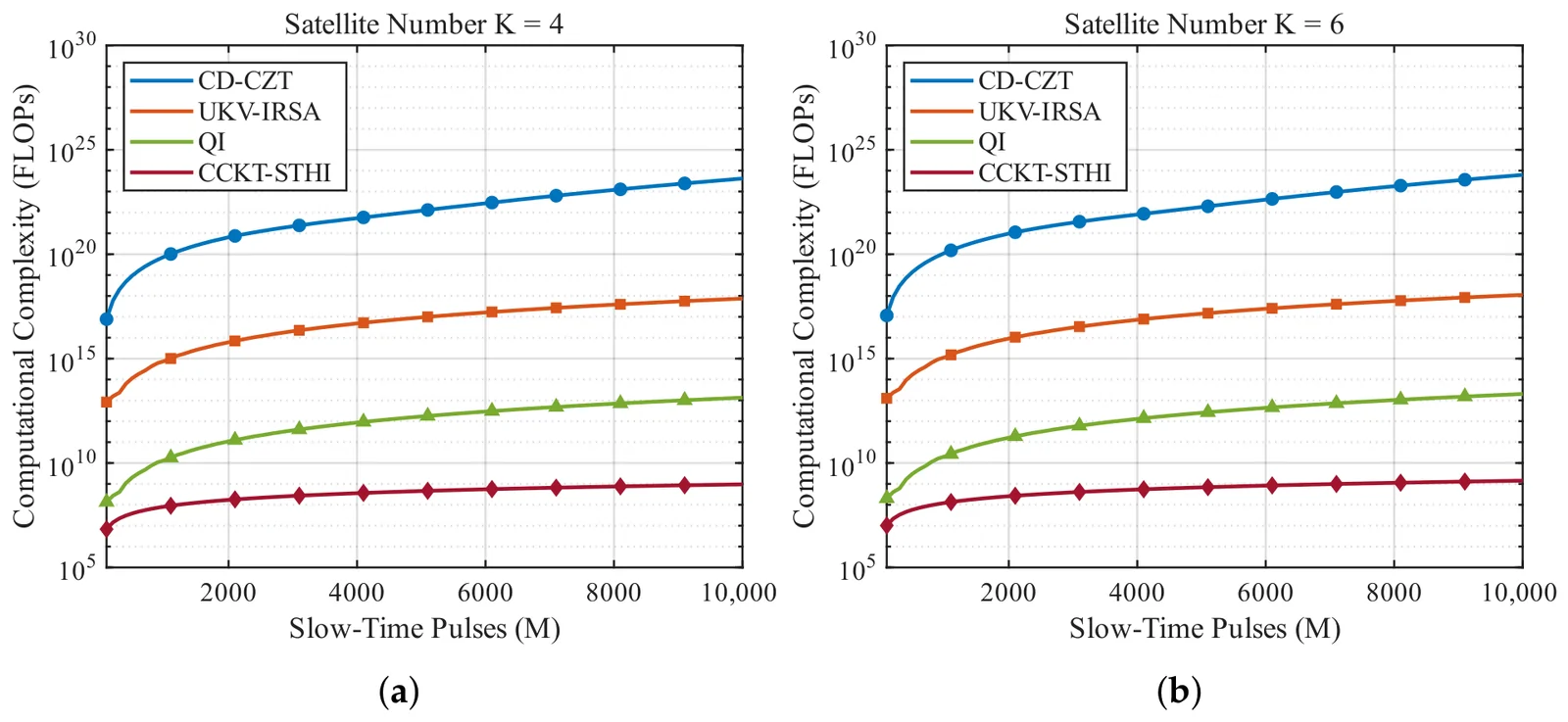
Floating-point operations (FLOPs) comparison with the search steps that keep the gain loss within 0.1 dB; the vertical axis is logarithmic. UKV–IRSA and CD-CZT use the search spans and steps given in Section 3.6, and the quadratic integrator (QI) and the centroid-compensated keystone transform-based space–time hybrid integration (CCKT-STHI), which are based on two-dimensional maritime-target models, are evaluated on the same azimuth and DFR grids without an elevation search. (**a**) Satellite number K = 4. (**b**) Satellite number K = 6.

Figure 7 compares the FLOP counts of UKV–IRSA, CD-CZT [32], QI [22], and CCKT-STHI [2] under the same observation parameters. QI and CCKT-STHI have lower computational costs because they employ two-dimensional maritime-target models. Both CD-CZT and UKV–IRSA include an elevation search and therefore require more FLOPs. However, UKV–IRSA replaces the two DOA-rate search dimensions and joint range–Doppler resampling of CD-CZT with a bounded DFR search and range-only IRSA. The search spans are ±15° in azimuth, ±5° in elevation, ±0.5°/s in each DOA rate and [−12.8,0] Hz/s in DFR, and the search steps keep the gain loss of each parameter within 0.1 dB. Both methods thus use DOA grids with approximately the same angular resolution, but CD-CZT needs about $5\times 10^{3}$ hypotheses for each DOA rate at $T_{n}=0.5$ s, whereas UKV–IRSA needs only three DFR hypotheses. Consequently, UKV–IRSA requires four to five orders of magnitude fewer FLOPs than CD-CZT for both K=4 and K=6.

Table 1 lists the runtimes of the two methods measured with MATLAB R2026a on the same computer for one 0.5-s frame of the four links (500 pulses and 1024 range cells). Both methods were implemented to perform the operations counted above; the time per search is the time required to evaluate one search hypothesis, and the total time is obtained by multiplying it by the number of hypotheses in the above search grids. A single UKV–IRSA search takes longer because it includes the coherent integration, DC estimation and IRSA of all links, but far fewer searches are required, so that the total search time of UKV–IRSA is about $5.0\times 10^{3}$ s, whereas that of CD-CZT is about $2.3\times 10^{9}$ s.

## 4. Simulation and Experimental Results

This section evaluates the proposed algorithm using numerical simulations and field experiments in two scenarios: an aircraft during takeoff and a short-range ground vehicle.

### 4.1. Simulation Results

In simulation experiments, four GPS satellites are configured as PMR illuminator sources. To achieve realistic bistatic geometry, their coordinates and velocities are matched to those derived from real ephemeris data. A passive receiver is positioned at XIDIAN University, collecting GPS L1 signals for target detection, and the target is set at a slant range of about 32.1 km from the radar. The coordinates are expressed in the local receiver-centered reference system, and the target coordinate in Table 2 is rounded for compactness.

**Figure 8:**
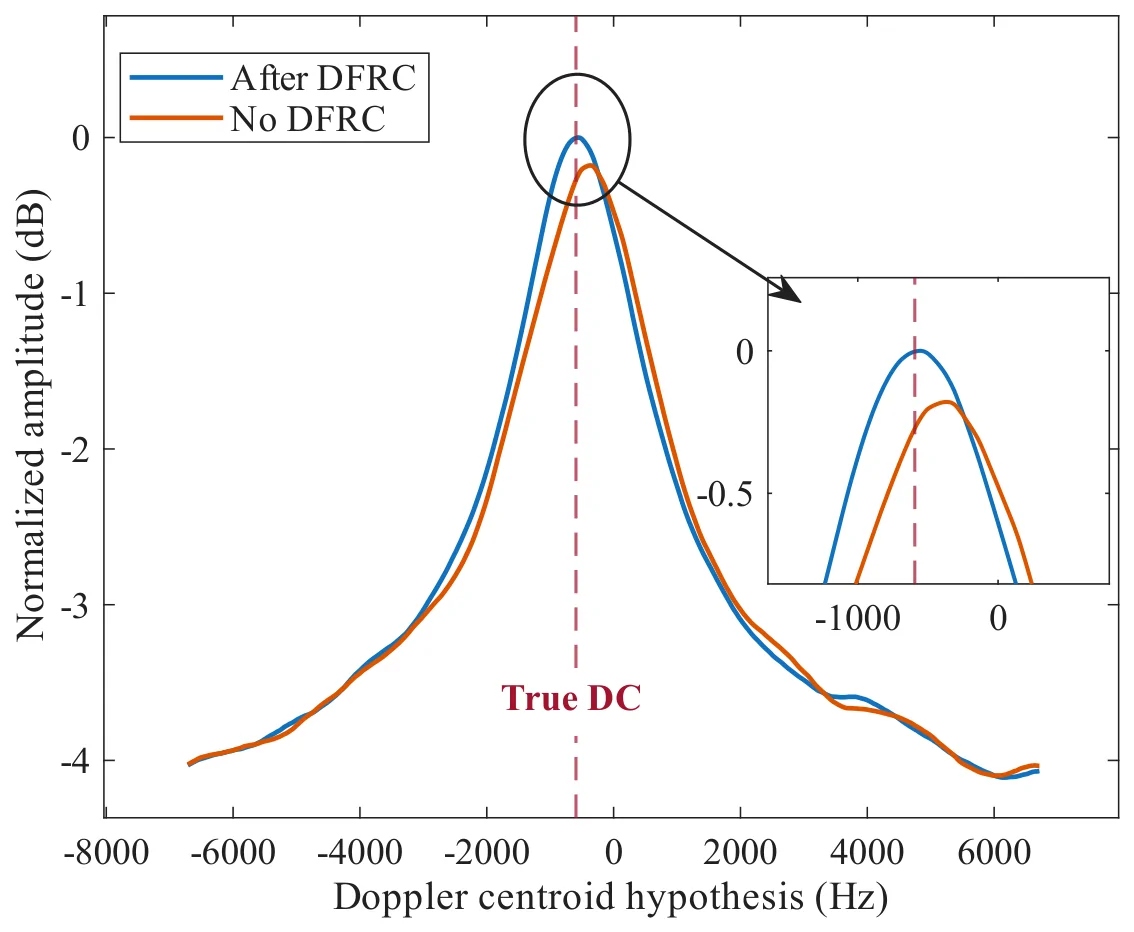
DC estimation result after DFRC processing.

The presence of DFR induces range curvature and Doppler migration, which impedes DC estimation and target energy accumulation. Due to the narrow signal bandwidth (i.e., 1.023 MHz of the GPS coarse/acquisition (C/A) code), the range resolution is coarse (about 147 m), so that the intra-frame range cell migration caused by the DFR is negligible; the focusing bottleneck instead lies in the Doppler dimension, where the link-dependent DC and the DFR must be accurately compensated to avoid Doppler spectral broadening and the attendant integration loss. Taking one bistatic link as an example, Figure 8 illustrates the effect of DFRC on DC estimation; both curves are normalized to the peak of the DFRC output and expressed in dB. Under the matched DFR hypothesis, the peak lies within about 37 Hz of the true DC indicated by the vertical dashed line, whereas without DFRC the peak is displaced by about 225 Hz and is 0.18 dB lower. The coherent integration time within each frame is set to $T_{n}=0.5$ s, consistent with the aircraft decorrelation interval analyzed in Section 2.

**Figure 9:**
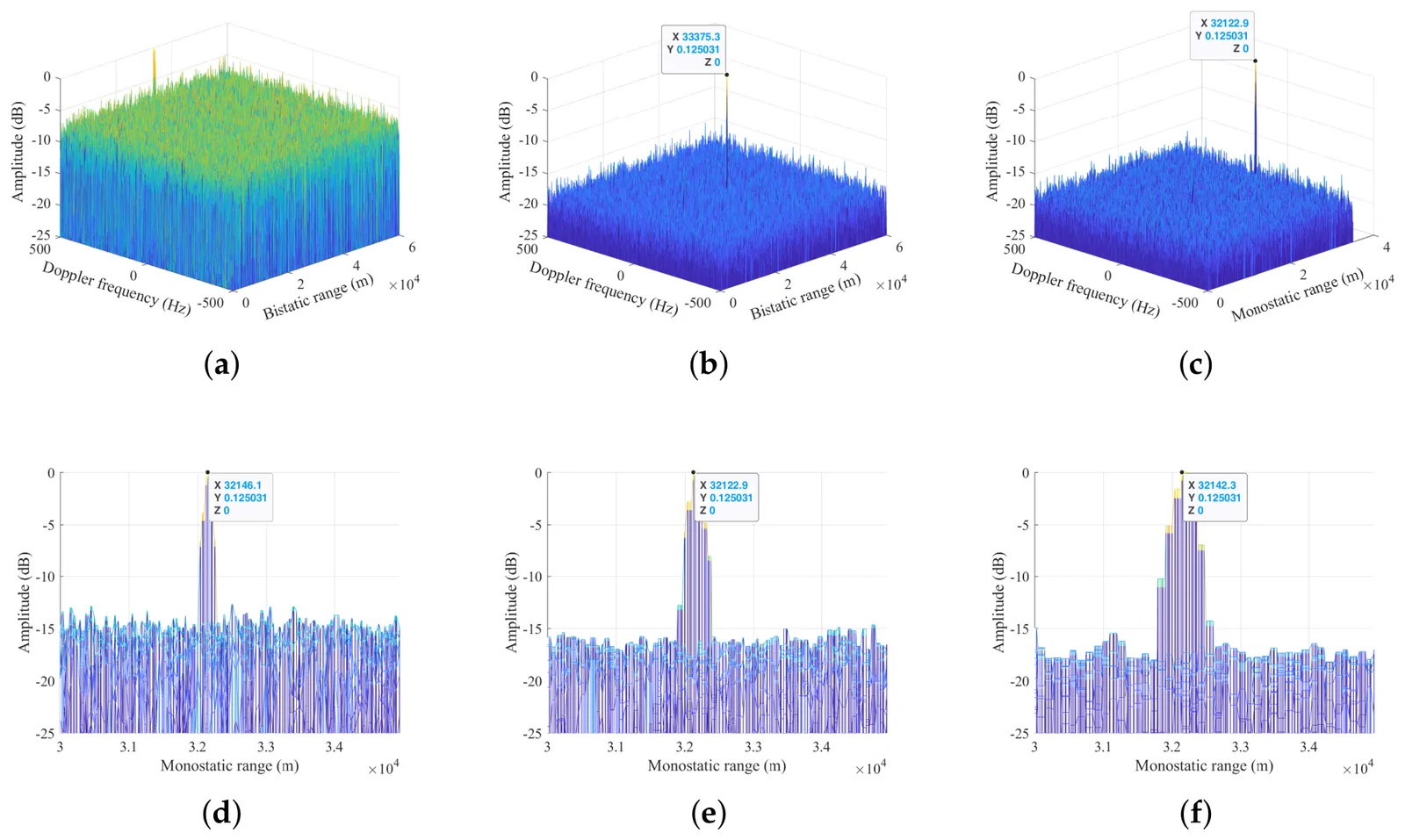
Coherent integration and IRSA results. (**a**–**c**) Satellite 3: BRD after moving target detection (MTD), BRD after DFRC and coherent integration, and MRD after IRSA. (**d**–**f**) MRD slices for Satellites 2–4.

Figure 9 summarizes the coherent integration and MRD-domain mapping results. For Satellite 3, uncompensated DC and DFR spread the target over multiple Doppler bins (Figure 9a), whereas DFRC and 0.5-s coherent integration refocus it into one Doppler bin and provide an approximately 27 dB peak-SNR gain (Figure 9b). IRSA then maps the response to the zero-Doppler MRD domain on a common range grid (Figure 9c), enabling cross-link fusion.

**Figure 10:**
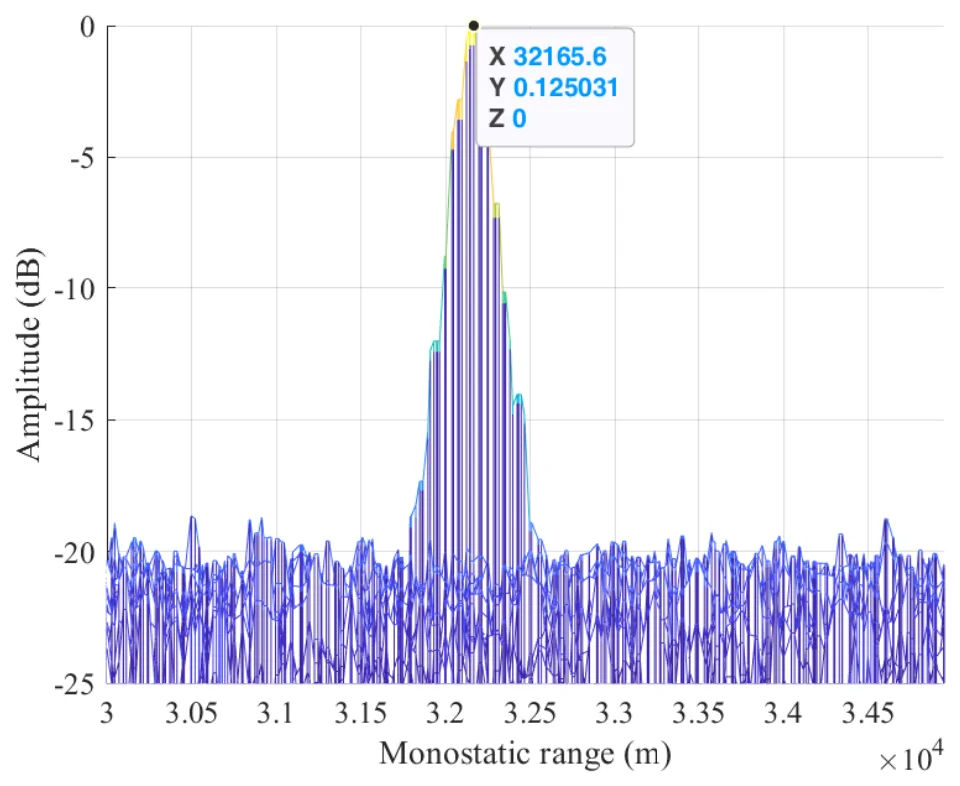
Fusion result of four satellites obtained by the proposed UKV–IRSA algorithm.

Figure 9d–f show the IRSA results for Satellites 2, 3, and 4. The target is aligned at the same MRD coordinate with a residual range error of about 10 m, while the response width varies with the link-dependent scaling factor ξ. This residual is well below the range resolution, because the DOA and the monostatic range are given by the location of the peak of the fused statistic in (39), which is estimated much more precisely than the peak width; it also lies within the quantization of the DOA step of 0.09°, which corresponds to a position step of about 50 m at 32.1 km. This alignment enables the multistatic fusion shown in Figure 10.

**Figure 11:**
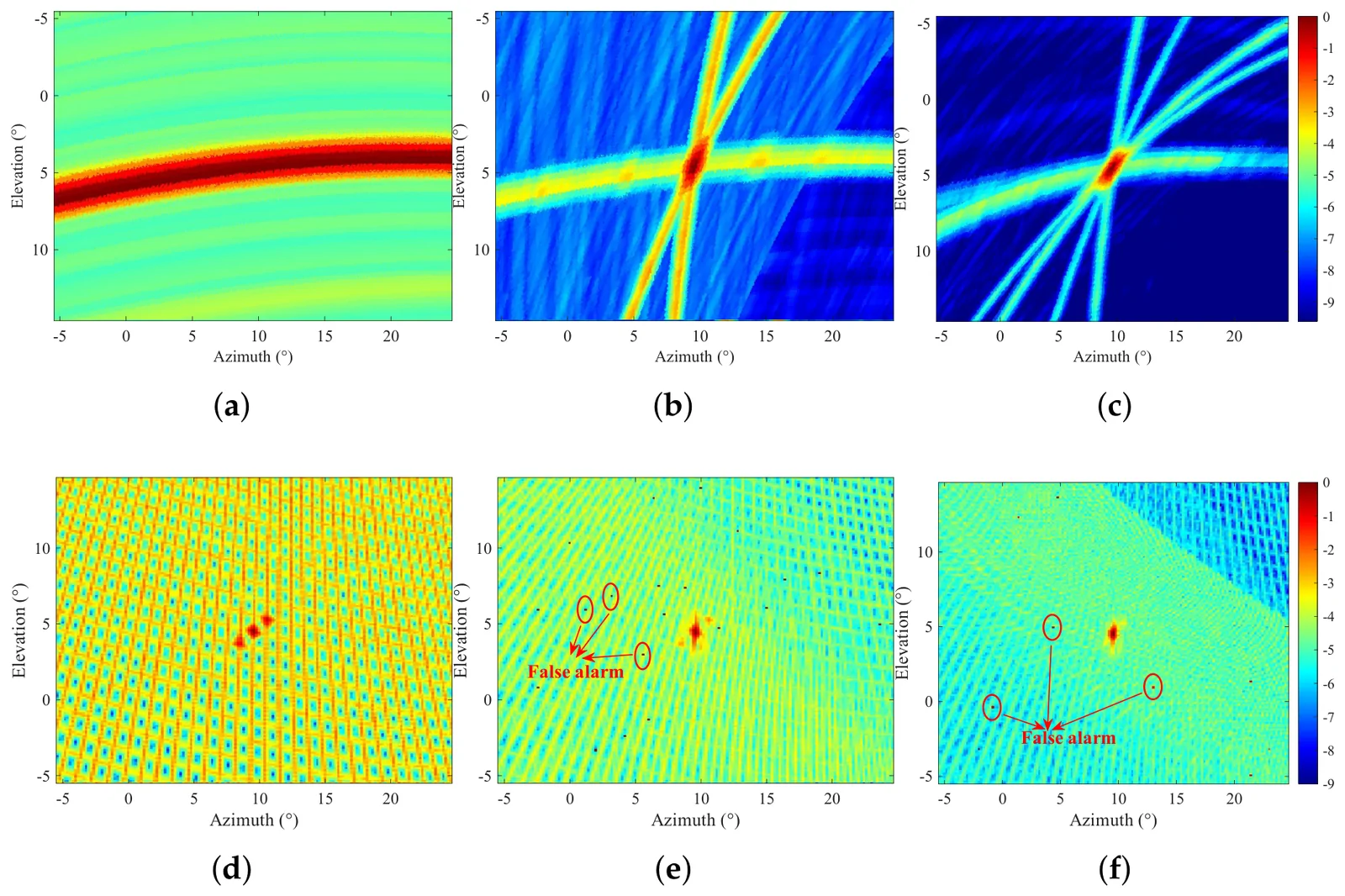
Comparison of DOA search maps (dB scale) under different satellite selections, obtained by the proposed and the CD-CZT methods; the horizontal and vertical axes are the azimuth and elevation hypotheses, respectively. (**a**) Satellite 1 and 2 under proposed method. (**b**) Satellite 1, 2 and 3 under proposed method. (**c**) Satellite 1, 2, 3 and 4 under proposed method. (**d**) Satellite 1 and 2 under CD-CZT method. (**e**) Satellite 1, 2 and 3 under CD-CZT method. (**f**) Satellite 1, 2, 3 and 4 under CD-CZT method.

Target localization is performed based on the DOA estimation result, and the number of selected satellites influences the localization outcome. Figure 11 presents the comparison of DOA estimation results for three different satellite configurations. Figure 11a shows the fusion result when satellites 1 and 2 are selected; as expected, two bistatic links provide an insufficiently constrained multistatic geometry, resulting in ambiguous 3D localization. In contrast, as shown in Figure 11b,c, the inclusion of satellite 4 leads to a noticeable improvement in the SNR of the target. Moreover, the target energy becomes concentrated into more confined resolution cells, thereby enhancing the localization accuracy. Finally, the DOA estimation is $\left({\hat{\phi }}_{tg}=9.57{}^{\circ},{\hat{\theta }}_{tg}=4.57{}^{\circ}\right)$. Figure 11d shows the DOA search result of CD-CZT method, under the same satellite illumination, CD-CZT can further narrow the angular range of the target compared to the proposed method, since its joint range–Doppler rescaling under the searched DOA rate imposes an additional constraint on the angle estimate, and Figure 11e,f similarly demonstrate comparable performance. However, it is difficult to maximize the aggregation of target energy because it is extremely sensitive to estimation errors in the DOA rate, making it challenging to effectively align the target in the Doppler dimension.

A key advantage of the proposed UKV–IRSA is that it rescales only the range axis when aligning the links: since the target Doppler has already been compensated to zero within each link during coherent integration, the Doppler axis is never rescaled. This removes the DOA-rate search and, more importantly, avoids the artifacts of joint range-Doppler rescaling. In CD-CZT, that joint rescaling—driven by the four-dimensional search over $\left(\theta_{tg},\phi_{tg},{\dot{\theta }}_{tg},{\dot{\phi }}_{tg}\right)$—is hypothesis-dependent, so a mismatched hypothesis pushes part of the Doppler spectrum out of band; the resulting truncation and unstable normalization create spurious peaks that raise false alarms and degrade the angular search.

Combined with the monostatic range measurement ${\hat{R}}_{r}^{\prime }=32.1656\;\mathrm{km}$, the receiver-centered target coordinate can be calculated as

(49) $$\begin{array}{c} {\hat{T}}_{tg}^{\mathrm{rel}}=\left[\begin{array}{c} 31.617\\ 5.331\\ 2.563 \end{array}\right]\;\mathrm{km} \end{array}$$

where the localization error is 25.3 m.

**Figure 12:**
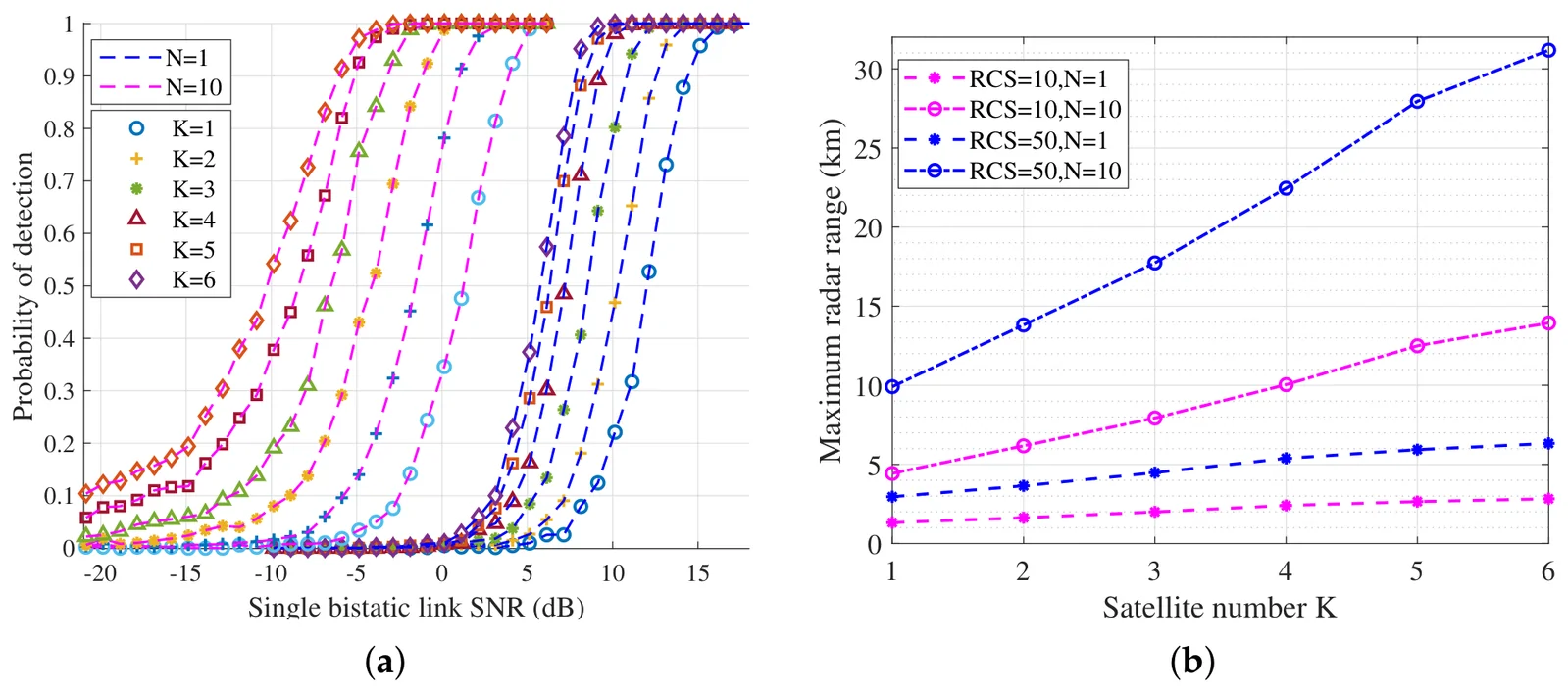
Performance analysis of PMR. (**a**) probability of detection comparison. (**b**) Maximum radar coverage.

Furthermore, to demonstrate the detection performance of the proposed UKV–IRSA method, two groups of Monte Carlo experiments (with the integration frame number *N* set as 1 and 10, respectively) are conducted. Specifically, for each group, the threshold is set to achieve the false alarm ratio of $10^{-4}$, and the SNR of each bistatic link is uniformly varied from −21 to 18 dB in steps of 1 dB (each SNR contains 100,000 independent trials). Here, the SNR is the single-link SNR after pulse compression and intra-frame coherent integration. As illustrated in Figure 12a, the results of N = 1 and N = 10 frames are represented as blue and pink lines, respectively. The six blue lines correspond to different satellite numbers *K*. For a single bistatic link, achieving a 90% detection probability requires a 14.4 dB SNR. Taking this single-link case (K=1) as the baseline, the required SNR is reduced by 1.8, 3.6, 5.2, 6.1, and 6.6 dB for K=2,3,4,5,6, respectively. When the integration frame number increases to 10, as shown by the pink lines, the required SNR is reduced by 13.3, 15.5, 17.5, 19.4, and 20.4 dB for K=2,3,4,5,6, respectively, relative to the same single-link, single-frame baseline. This indicates that the PMR system can achieve a larger surveillance range than the PBR system; alternatively, at the same range, it can detect aircraft with a smaller RCS, such as UAVs. Figure 12b shows the corresponding maximum surveillance coverage of PMR for aircraft with different satellite number *K* and integration frame number *N*, respectively. Considering forward-scattering gain [11], two RCS values of $10\;m^{2}$ and $50\;m^{2}$ are considered. The equivalent isotropic radiated power (EIRP) of GNSS satellites is set as 33 dBW [38], antenna gain as 20 dB; with 5 dB of system losses. The results show that the proposed UKV–IRSA method clearly enhances the coverage, enabling aircraft detection over a range of several tens of kilometers.

**Figure 13:**
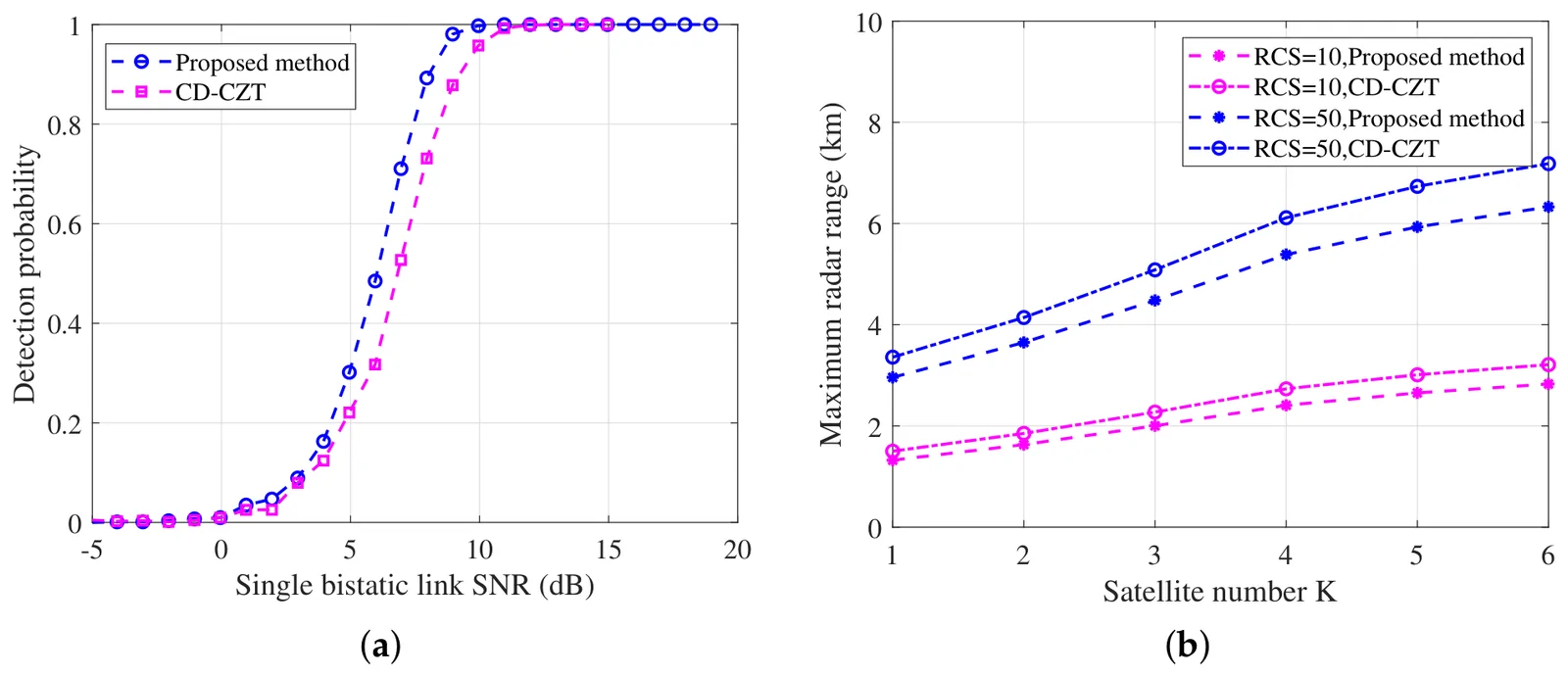
Performance comparison of the proposed method and the CD-CZT method. (**a**) Probability of detection comparison. (**b**) Maximum radar coverage comparison.

**Figure 14:**
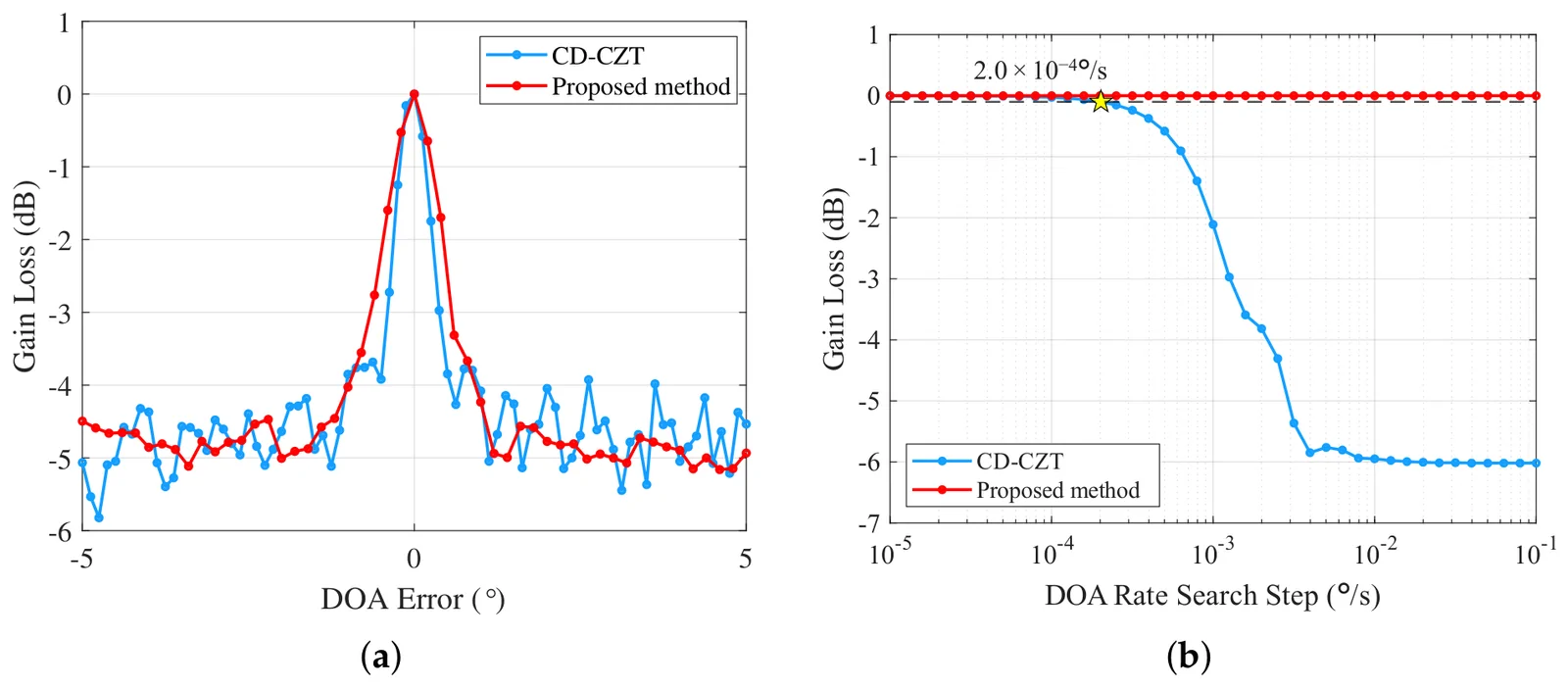
Integration gain loss versus the parameter error and the search step. (**a**) Gain loss with respect to DOA offset. (**b**) Gain loss with respect to the DOA-rate search step.

Another series of simulations is conducted for performance comparison with frame number N=1 and satellite number K=4, as shown in Figure 13. Compared with the CD-CZT method, the proposed method reduces the SNR threshold by 1.1 dB at a detection probability of 0.9. This improvement increases the maximum detection range of the radar by 14% and 13% for target RCS of $10\;m^{2}$ and $50\;m^{2}$, respectively. To better highlight the effect of parameter estimation error on the integration gain loss, Figure 14 illustrates the integration gain loss as a function of the DOA offset between the tested and actual values and of the DOA-rate search step when all four satellites are used as transmitters. Under the same 0.1 dB loss and $T_{n}=0.5$ s, both UKV–IRSA and CD-CZT require a DOA search step of 0.09°, and CD-CZT additionally requires a DOA-rate search step of $2.0\times 10^{-4}$°/s; these steps are used for both methods in the simulation comparison and in the aircraft field experiment to ensure a fair comparison. For coarser DOA-rate steps, the gain loss of CD-CZT increases rapidly and reaches 6 dB at the step of about 0.06°/s that CD-CZT can afford with the FLOPs of UKV–IRSA. In contrast, the proposed UKV–IRSA method estimates the link-dependent target Doppler through RFT-based coherent integration and therefore eliminates the need for high-precision DOA-rate estimation. Consequently, the proposed method is essentially insensitive to the DOA-rate search step in Figure 14b, because it does not use the DOA rate as a search variable.

### 4.2. Experimental Results

To verify the performance of the proposed algorithm across different satellite geometries, target categories, and motion states, two field experiments are presented in this section.

#### 4.2.1. Scenario 1: Aircraft Takeoff

**Figure 15:**
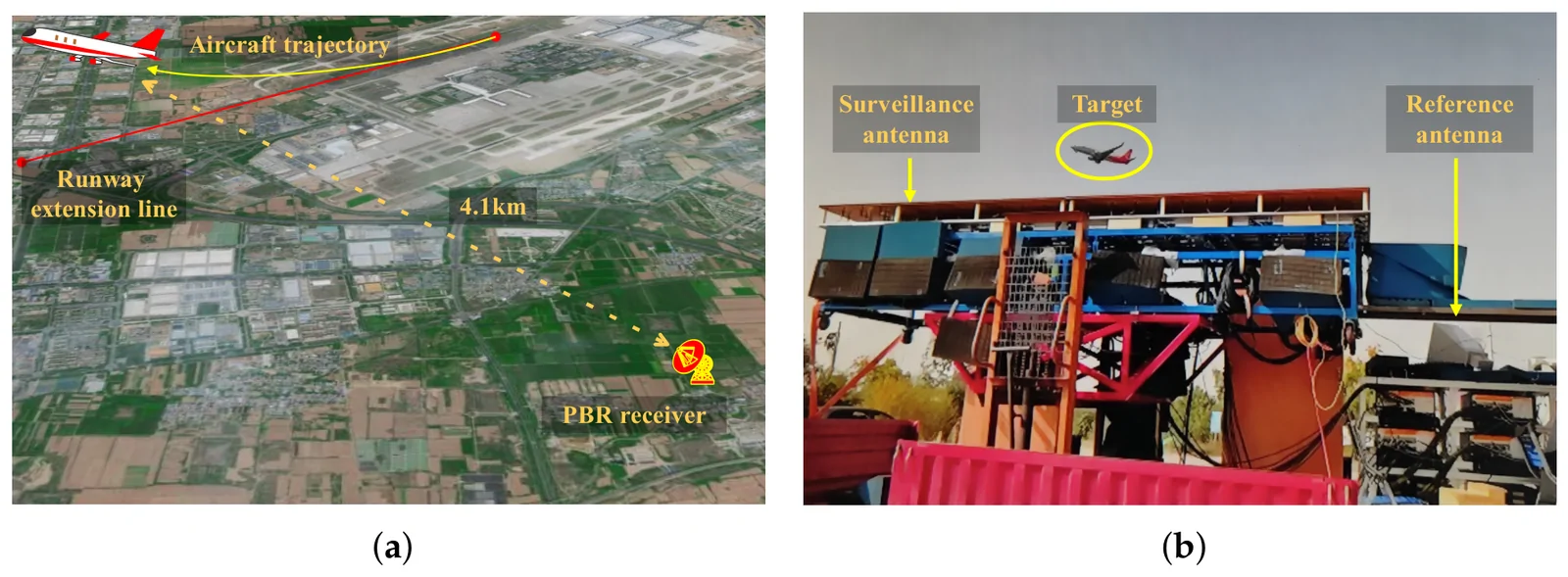
Aircraft field experiment. (**a**) Detection geometry. (**b**) Photograph of the system setup.

**Figure 16:**
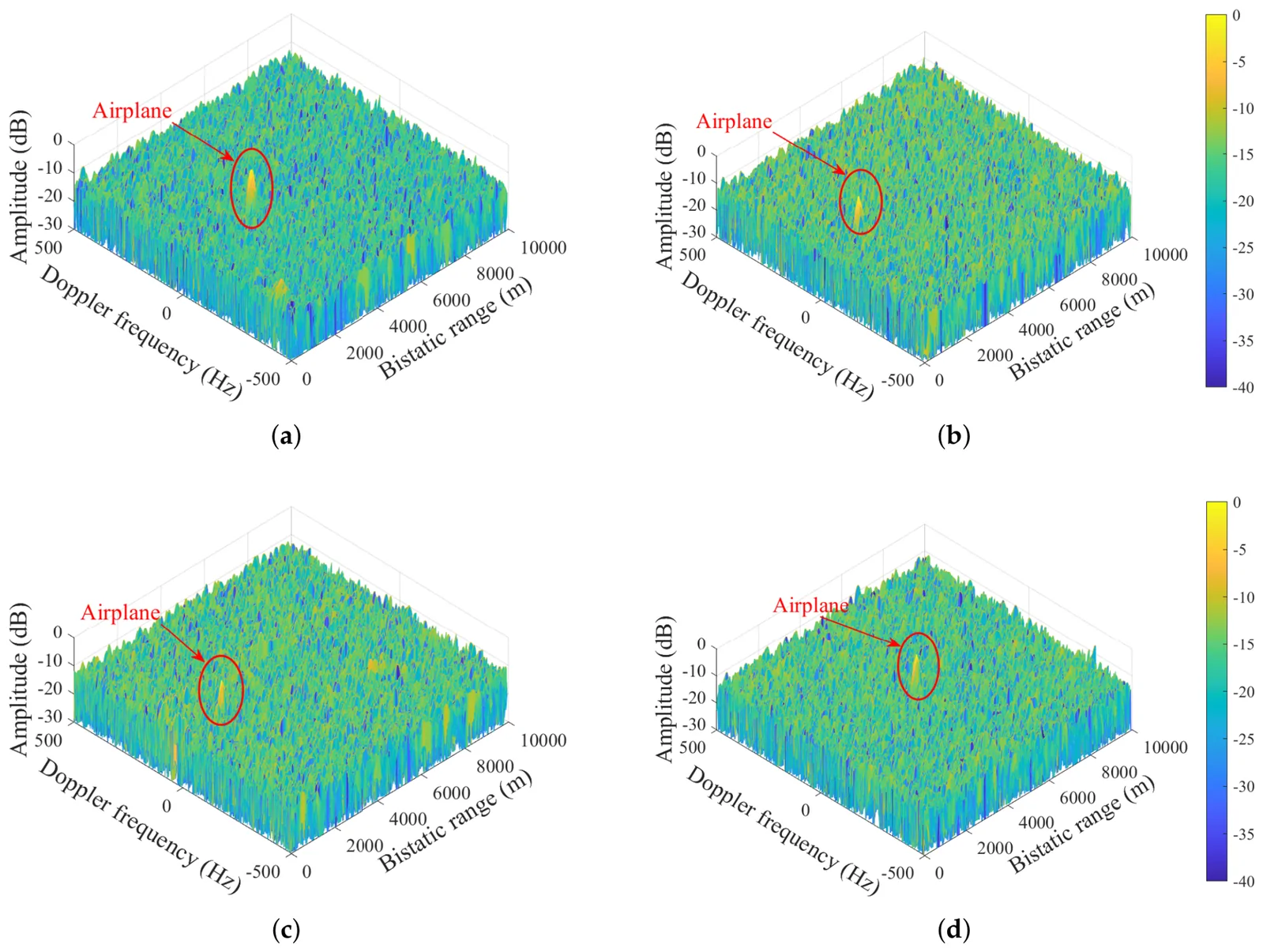
Comparison of integration results in the BRD domain before IRSA. (**a**) PRN 15. (**b**) PRN 18. (**c**) PRN 29. (**d**) PRN 24.

**Figure 17:**
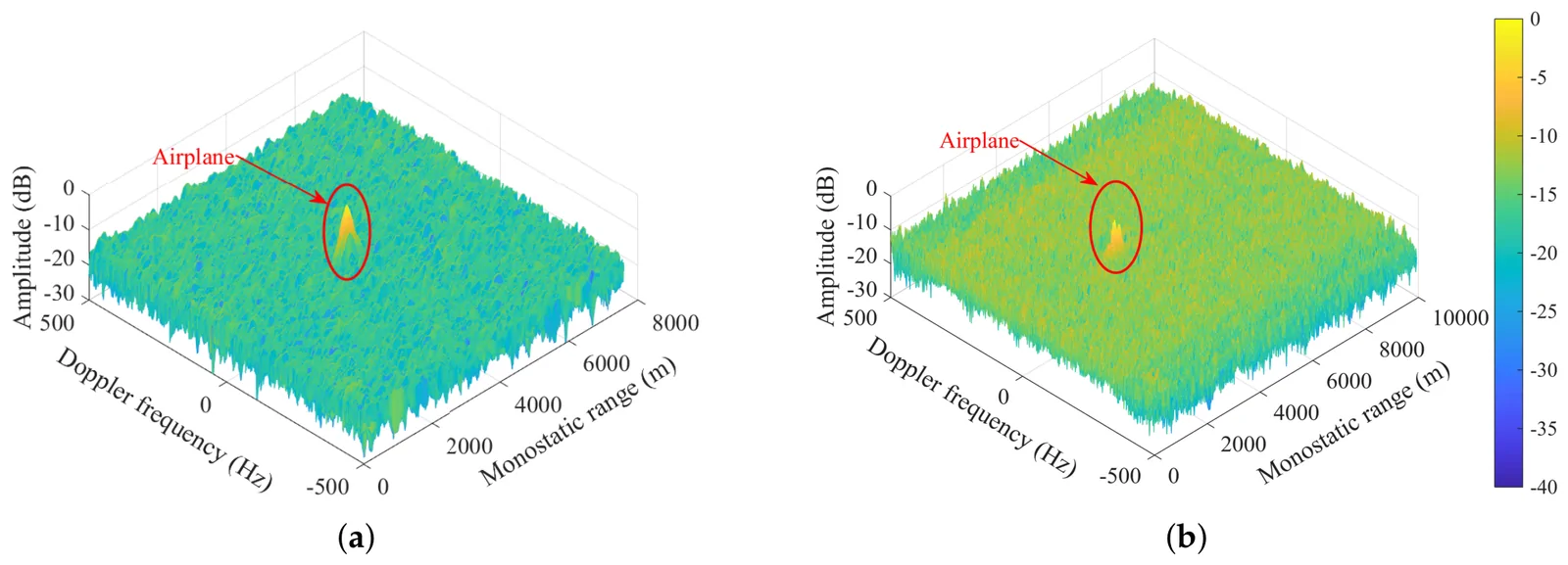
Measured-data MRD fusion results. (**a**) UKV–IRSA. (**b**) CD-CZT.

To further corroborate the efficacy of the proposed approach under realistic operational conditions, a field measurement campaign was conducted at Xi’an Xianyang International Airport. Four GPS satellites operating on the L1 band (1575.42 MHz), with PRNs 15, 18, 29, and 24, were selected as illuminators in the PMR configuration; it should be noted that the proposed method is equally applicable to any arbitrary subset of visible GNSS satellites. The principal experimental parameters are compiled in Table 3. The detection geometry of the PMR experiment is schematically depicted in Figure 15a. A civil aircraft during takeoff was chosen as the target of opportunity, and a photograph capturing both the target aircraft and the radar receiver setup is presented in Figure 15b. The collected signals from both the reference and surveillance channels are down-converted to baseband, where the reference channel signal is decoded via a software GNSS receiver, enabling both satellite and receiver positioning. The reference and surveillance channels share a common local oscillator and sampling clock, ensuring that the two channels are inherently synchronized in both time and frequency domains. Figure 16 displays the BRD maps accumulated across four frames, accounting for RCS fluctuation, with each frame subjected to 0.5 s of coherent integration. The target’s Doppler centroid is accurately estimated and compensated to zero frequency. To quantitatively assess the effectiveness of the proposed algorithm, the accumulated target energy is extracted from the measured peak response. With a contiguous segment of noise samples as the noise reference, the single-link SNRs measured on the four-frame accumulated BRD maps (i.e., after pulse compression, coherent integration within each frame, and noncoherent accumulation over the four frames) are 11.5, 9.7, 9.5, and 9.9 dB for the four satellite links, respectively. Figure 17 compares the MRD fusion results obtained from the measured data. UKV–IRSA effectively concentrates the target response and achieves a fused SNR of 14.7 dB, representing a 3.2-dB improvement over the strongest individual link. In contrast, the CD-CZT response remains noticeably broadened along the Doppler dimension, as DOA-rate estimation errors introduce residual cross-link misalignment that prevents full energy focusing.

**Figure 18:**
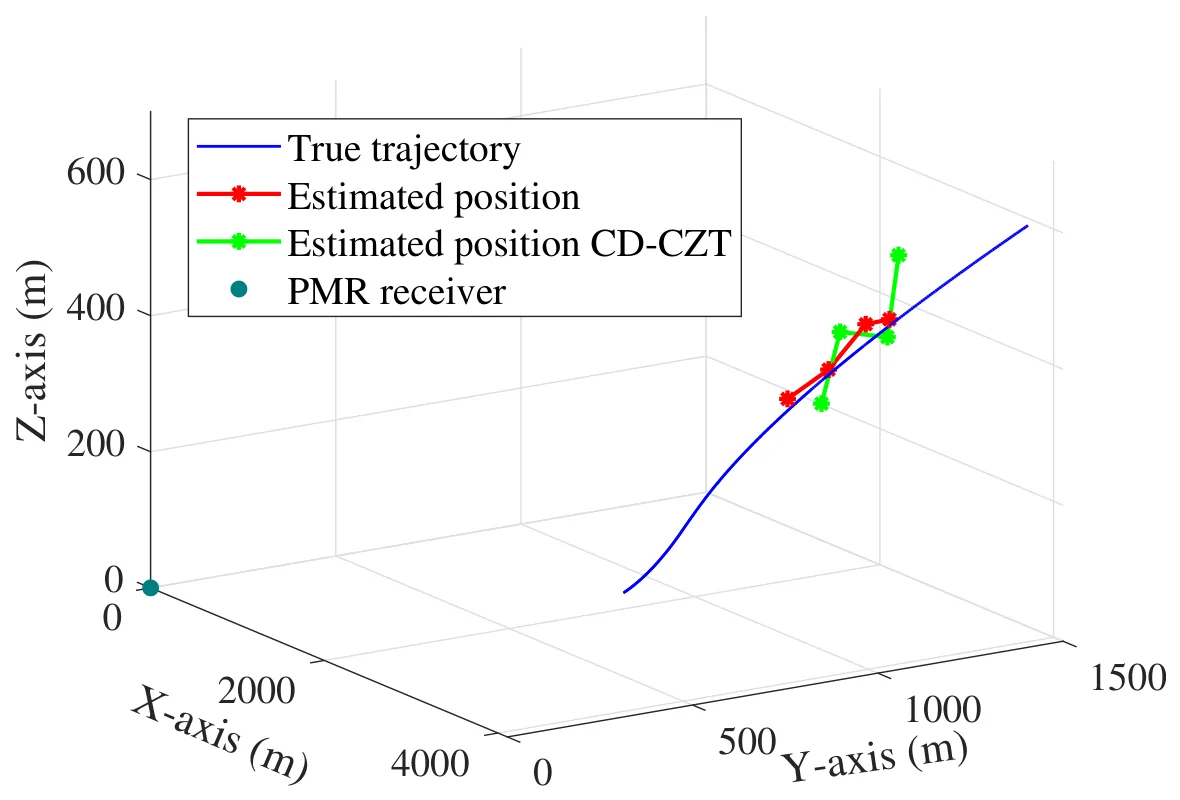
Reference and estimated aircraft trajectories.

Using satellite ephemerides, the measured DOA, and monostatic range, the target tracks are estimated in receiver-centered coordinates (Figure 18). The independent reference trajectory is recorded by an onboard multi-GNSS logger with meter-level uncertainty. UKV–IRSA closely follows this reference with a root-mean-square error (RMSE) of 56.9 m, whereas CD-CZT shows larger deviations; the quantitative comparison is given in Table 4. At the target range of about 4.1 km, the DOA step of 0.09° corresponds to a position step of about 6.4 m, so the localization error is dominated by noise and by the geometric amplification of the per-link range errors.

#### 4.2.2. Scenario 2: Short-Range Ground Vehicle

**Figure 19:**
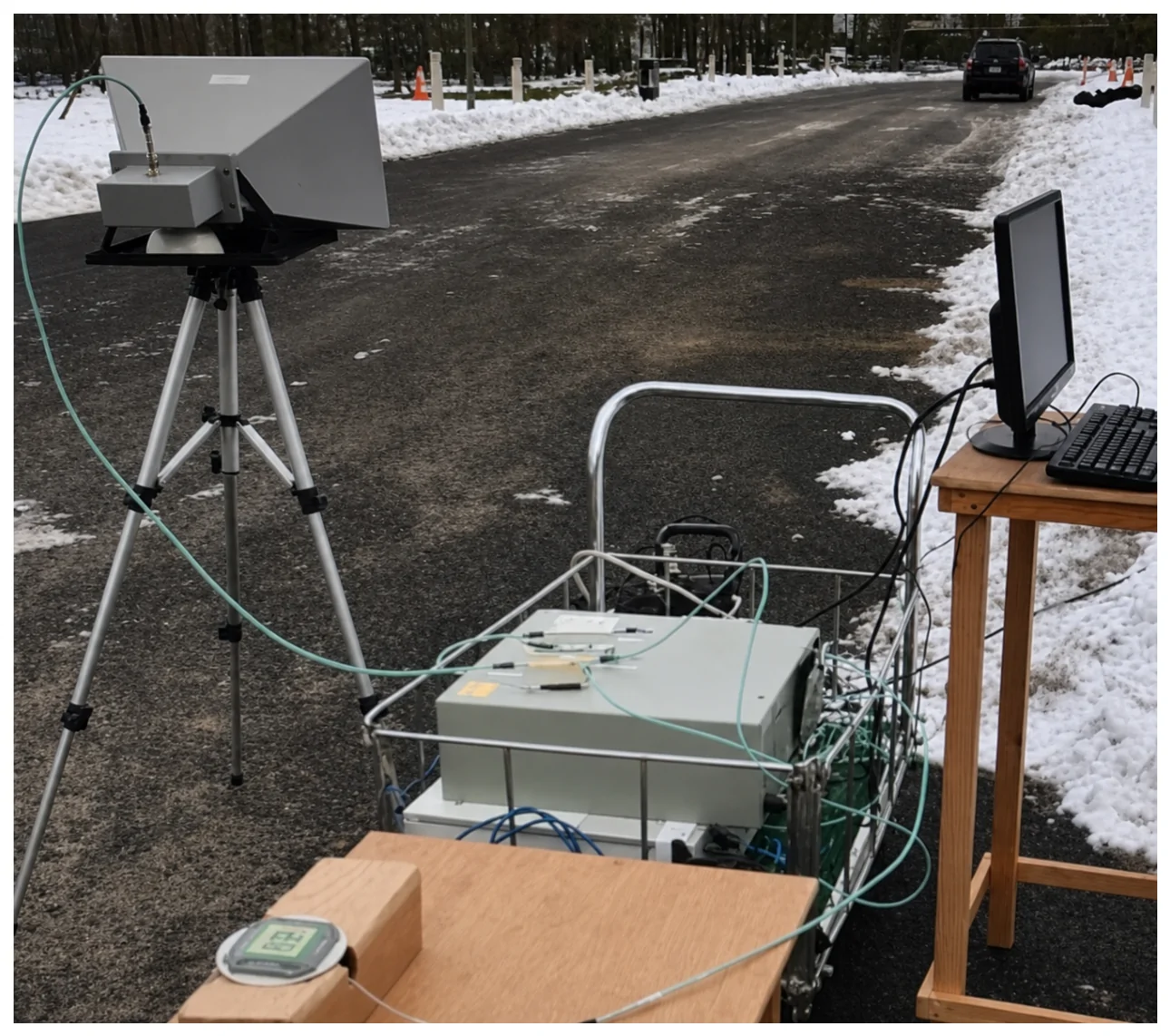
Ground-vehicle field experiment: receiver setup and target vehicle.

In the second experiment, UKV–IRSA was evaluated with a slow-moving ground vehicle at short range. The receiver setup and target vehicle are shown in Figure 19. In this experiment, four GPS L1 satellites with PRNs 5, 13, 18, and 22 were used as illuminators. The vehicle approached the stationary receiver along a straight road at approximately 5 m/s from an initial separation of approximately 100 m. The nominal initial azimuth and elevation were both 0°, with azimuth measured from east towards north. The measured surveillance signal was cross-correlated with the direct-path C/A reference for each illuminator, and one 0.8-s coherent frame containing 800 C/A-code periods was processed.

**Figure 20:**
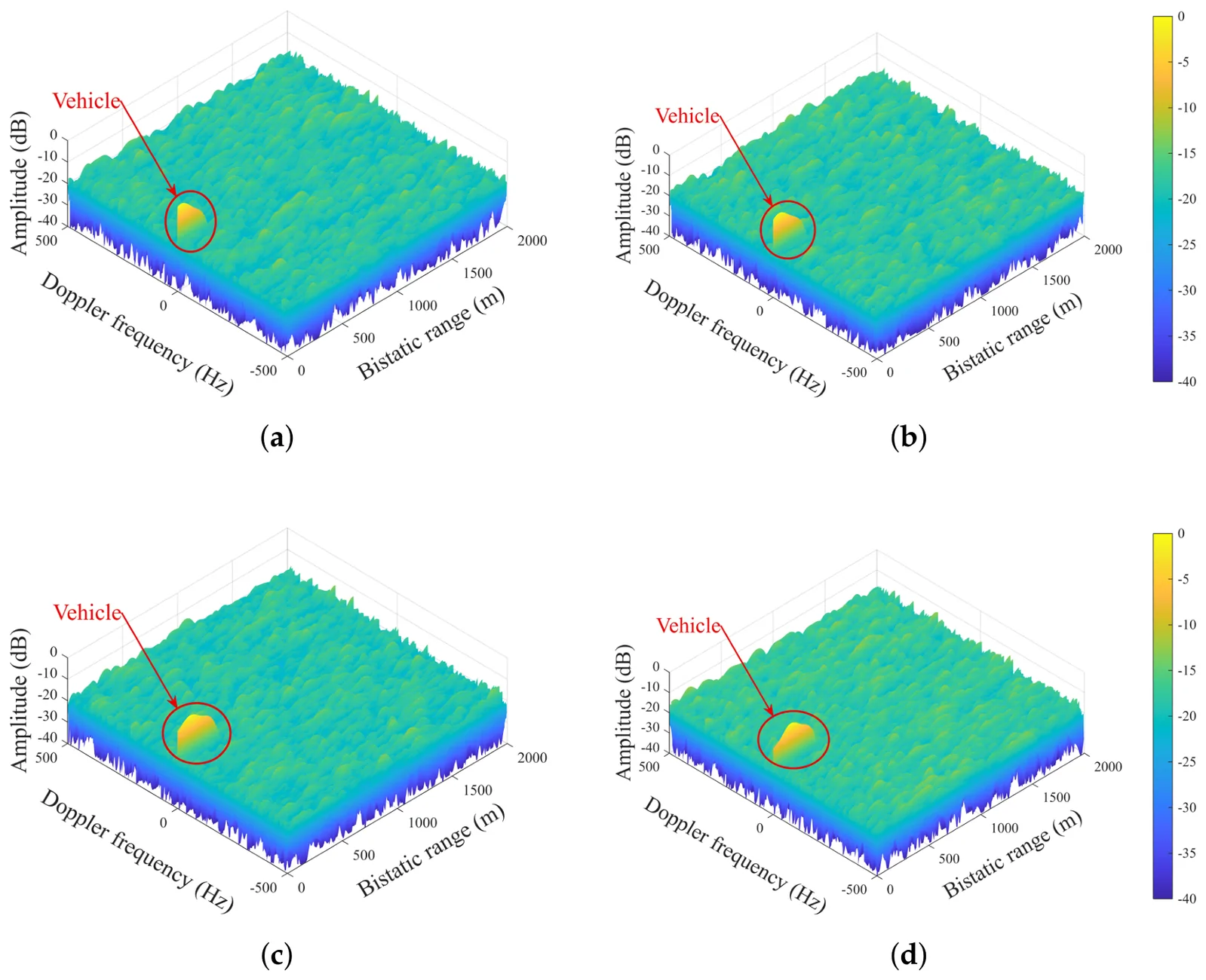
Measured BRD maps of the ground vehicle after DFRC and 0.8-s coherent integration, with the estimated DC compensated to zero frequency. (**a**) PRN 5. (**b**) PRN 13. (**c**) PRN 18. (**d**) PRN 22.

Figure 20 presents the measured BRD maps after DFRC and 0.8-s coherent integration, with the estimated DC compensated to zero frequency in each link. The vehicle responses peaked at bistatic ranges of approximately 30, 57, 112, and 172 m for PRNs 5, 13, 18, and 22, respectively, illustrating the link-dependent range signatures before IRSA.

**Figure 21:**
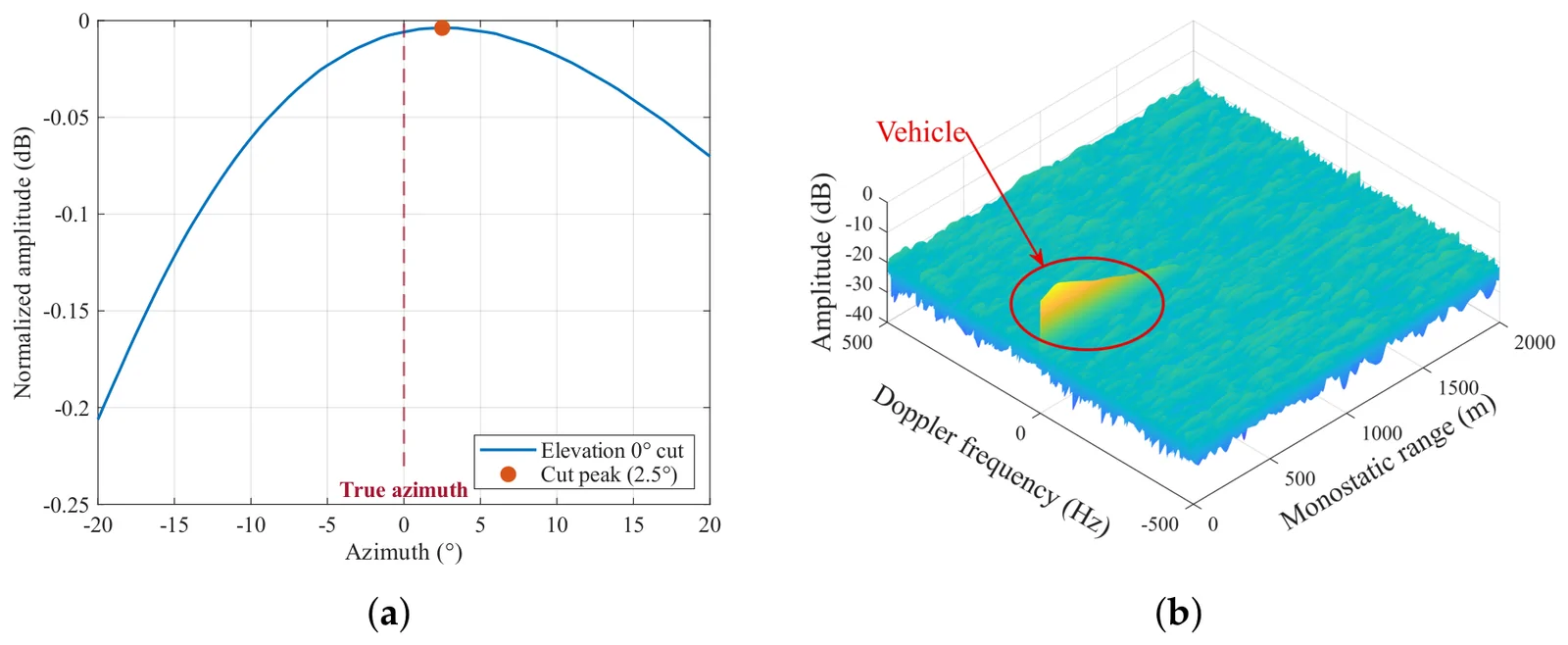
Ground-vehicle results obtained from measured data. (**a**) Zero-elevation DOA cut. (**b**) MRD map fused by UKV–IRSA.

Figure 21 shows the zero-elevation DOA cut and the fused MRD map. UKV–IRSA aligns the link responses at a common monostatic range of about 105 m and at zero Doppler, and the fusion reduces the coefficient of variation of the background power from 1.0 to 0.51.

The detection probabilities in Table 5 were calculated from the theoretical detection probability of a nonfluctuating target under square-law detection [31], evaluated at the local SNRs estimated from the BRD maps in Figure 20 (7.7, 7.3, 8.1, and 6.8 dB for PRNs 5, 13, 18, and 22, respectively), which were assumed to represent the average values; for the fused map, the noise-normalized responses of the four links were summed noncoherently. Table 5 shows that UKV–IRSA achieved higher detection probabilities than all individual links at the three false-alarm settings. At $P_{\mathrm{fa}}=10^{-4}$, the detection probability increased from 27.9% for the strongest individual link (PRN 18) to 94.9% after fusion, an improvement of 67.0 percentage points. At $P_{\mathrm{fa}}=10^{-5}$, the fused detection probability remained 87.5%, demonstrating improved vehicle detectability through multi-illuminator fusion. Because the fusion reduces the background fluctuation, the threshold for a given false-alarm probability is lower relative to the mean noise power, so that high detection probabilities are retained even at low false-alarm probabilities.

## 5. Discussion

The numerical simulations and the two field experiments support the effectiveness of UKV–IRSA for multi-illuminator integration. The method coherently integrates each bistatic link along a UKV-constrained trajectory, remaps the integrated responses onto a shared monostatic-range–zero-Doppler domain via range-axis scaling, and performs noise-normalized noncoherent fusion across both illuminators and frames. Because the target energy is already focused at zero Doppler within each link prior to alignment, neither DOA-rate search nor Doppler-axis resampling is required. This formulation judiciously exploits the common DOA and the dominant DFR component while preserving the full link-specific Doppler histories.

The detection results vividly demonstrate the synergistic benefit of harnessing both spatial and temporal integration resources. At a detection probability $P_{d}=0.9$, six-link integration reduces the required single-link SNR by 6.6 dB for a single frame and by 20.4 dB for ten frames, both measured relative to the single-link, single-frame baseline. The four-link aircraft field experiment achieves a fused SNR of 14.7 dB–3.2 dB higher than the strongest individual link—and delivers a localization RMSE of 56.9 m. These findings establish that the visible GNSS constellation overhead constitutes a substantial additional spatial energy resource that simultaneously enhances target detectability and provides geometric diversity for improved localization accuracy.

Prior multi-illuminator approaches have primarily addressed two-dimensional maritime target motion or relied on a common DFR or DOA-rate estimate [2,21,22]. UKV–IRSA extends the scope of multi-illuminator integration to fully three-dimensional aircraft motion and eliminates the four-dimensional DOA/DOA-rate search demanded by CD-CZT [32]. Its common-domain response additionally enables joint detection and localization from a single passive receiving station, offering a readily deployable and covert surveillance architecture that requires no dedicated transmitter infrastructure.

The experiments serve to validate the underlying processing mechanisms rather than to exhaustively characterize every conceivable satellite–target–receiver geometry. Satellite coordinates are not treated as fitted parameters; instead, they are extracted directly from decoded ephemerides and used to recompute the link-specific trajectories and IRSA scaling factors. The simulation matrix encompasses range-axis expansion, near-unity mapping, and contraction regimes, while the mismatch and Monte Carlo experiments systematically vary estimation errors, SNR, link count, and frame count. The aircraft field campaign further employed four actual GPS L1 links with diverse geometries and measured per-link SNRs ranging from 9.5 to 11.5 dB. The aircraft results support focusing, registration, fusion, and localization under the stated assumptions, while the vehicle results support detection and range registration in a geometry with weak angular observability. Performance therefore depends on satellite geometry, visibility, synchronization, and parameter observability.

The second-order frame model treats the frame-center DC and DFR as local coefficients and therefore also describes accelerated motion, provided that the frame-center DFR is covered by the DFR search and the DFR remains approximately constant within a frame. For $T_{n}=0.5$ s and a target range of 32.1 km, limiting the coherent-power loss caused by the within-frame DFR drift to 0.1 dB yields conservative acceleration limits of about 140 m/s^2^ at the target speed of Table 2 (99 m/s) and 84 m/s^2^ at 200 m/s; modest maneuvers are thus covered, whereas stronger maneuvers may require shorter frames or higher-order compensation. In multi-target scenarios, an erroneous UKV hypothesis may associate responses that originate from different targets across different satellite links. Because link-wise Doppler peak selection and compensation align each of these responses to approximately zero Doppler, the subsequent IRSA registration and noncoherent fusion can produce spurious ghost peaks. Constraints based on a common DFR and on Doppler consistency suppress such inconsistent associations, yet they cannot guarantee their rejection. A more decisive remedy is to discriminate the interfering components before applying a generalized likelihood ratio test; this two-stage strategy has been shown to suppress related ghost targets in multisite radar [39] and therefore provides a principled basis for extending UKV–IRSA to multi-target operation. Future investigations will extend validation to additional GNSS constellations, geometric configurations, target trajectories, moving-receiver scenarios, and multitarget ghost-rejection algorithms.

## 6. Conclusions

This paper presents UKV–IRSA for the joint detection and localization of moving aircraft using multiple GNSS illuminators and a single passive receiver. The UKV provides a link-decoupled representation of target geometry to constrain coherent integration within each bistatic link, while IRSA registers the integrated responses in a common domain through range-axis scaling. This formulation avoids DOA-rate estimation and the associated error propagation. Noise-normalized noncoherent fusion then combines the aligned responses across illuminators and frames. Numerical simulations and two field experiments validate coherent integration and multi-illuminator fusion under the stated assumptions. The aircraft experiment further demonstrates three-dimensional localization, whereas the short-range vehicle experiment shows that angular observability can limit position estimation even when the target is detected. These results demonstrate the value of combining array-derived spatial information with multi-illuminator signal processing for remote sensing and target localization. By using visible GNSS illuminators as additional spatial integration resources, the proposed approach extends the integration gain beyond single-illuminator operation without dedicated transmit infrastructure. Future work will extend the validation to broader satellite geometries, moving-receiver platforms, and multitarget surveillance scenarios.

## Figures and Tables

**Table 1:** Measured time per search and estimated total search time of UKV–IRSA and CD-CZT for one 0.5-s frame of four links.

Method	Time per Search (ms)	Number of Searches	Total Time (s)
UKV–IRSA	45	$1.1\times 10^{5}$	$5.0\times 10^{3}$
CD-CZT	2.5	$9.2\times 10^{11}$	$2.3\times 10^{9}$

**Table 2:** Simulation parameters.

Parameters		Unit	Value
Satellite 1	PRN	-	13
	Signal frequency	MHz	1575.42
	Coordinate	km	(−12,219, 2763, 17,874)
	Velocity	m/s	(−389, −2710, 808)
Satellite 2	PRN	-	5
	Signal frequency	MHz	1575.42
	Coordinate	km	(−17,751, 2098, 13,586)
	Velocity	m/s	(−2081, −612, −2056)
Satellite 3	PRN	-	15
	Signal frequency	MHz	1575.42
	Coordinate	km	(−4195, 13,435, 14,155)
	Velocity	m/s	(−1156, −2027, 1784)
Satellite 4	PRN	-	18
	Signal frequency	MHz	1575.42
	Coordinate	km	(4760, 10,861, 17,499)
	Velocity	m/s	(−1959, 1753, −1066)
Target	Coordinate	km	(31.593, 5.336, 2.560)
	Velocity	m/s	(50, −80, 30)
Receiver	Coordinate	km	(0, 0, 0)
	Velocity	m/s	(0, 0, 0)

**Table 3:** Experimental parameters of the aircraft scenario.

Parameter	Unit	Value
Illuminator	-	GPS L1
Carrier frequency	MHz	1575.42
Satellite PRNs	-	15, 18, 29, 24
Signal bandwidth (C/A)	MHz	1.023
Coherent frame length $T_{n}$	s	0.5
Number of frames *N*	-	4
Total aperture time $T_{a}$	s	2
Target	-	civil aircraft
Receiver site	-	Xianyang Airport
Reference logger	-	Dragy Pro

**Table 4:** Experimental performance comparison for the aircraft scenario.

Metric	Proposed Method	CD-CZT Method
Integrated SNR (dB)	14.7	13.2
Localization RMSE (m)	56.9	121.0

**Table 5:** Vehicle detection probabilities before and after UKV–IRSA fusion at different false-alarm probabilities.

$P_{\mathrm{fa}}$	PRN 5	PRN 13	PRN 18	PRN 22	UKV–IRSA Fusion
$10^{-3}$	44.7%	38.8%	50.0%	31.8%	98.6%
$10^{-4}$	23.6%	19.2%	27.9%	14.5%	94.9%
$10^{-5}$	10.9%	8.3%	13.6%	5.8%	87.5%

## Data Availability

The data are not publicly available due to confidentiality constraints related to the radar system.

## Abbreviations

The following abbreviations are used in this manuscript:

BRD	Bistatic Range–Doppler
BTB	Back to Basics
C/A	Coarse/Acquisition
CCKT-STHI	Centroid-Compensated Keystone Transform-Based Space–Time Hybrid Integration
CD-CZT	Common-Domain Chirp Z-Transform
CZT	Chirp Z-Transform
DC	Doppler Centroid
DFM	Doppler Frequency Modulation
DFR	Doppler Frequency Rate
DFRC	Doppler Frequency-Rate Compensation
DOA	Direction of Arrival
DPI	Direct-Path Interference
ECA-BS	Extensive Cancellation Algorithm–Batches and Subbands
EIRP	Equivalent Isotropic Radiated Power
FFT	Fast Fourier Transform
FLOPs	Floating-Point Operations
GLONASS	Globalnaya Navigatsionnaya Sputnikovaya Sistema
GNSS	Global Navigation Satellite System
GPS	Global Positioning System
IFFT	Inverse Fast Fourier Transform
IRSA	Interlink Range Scaling Alignment
LOS	Line of Sight
MC	Multistatic Compensation
MMT	Maritime Moving Target
MRD	Monostatic Range–Doppler
MSCFT	Modified Scaled Fourier Transform
MTD	Moving Target Detection
MTI	Moving Target Indication
PBR	Passive Bistatic Radar
PC	Pulse Compression
PMR	Passive Multistatic Radar
PRI	Pulse Repetition Interval
PRN	Pseudorandom Noise
QI	Quadratic Integrator
RCM	Range Cell Migration
RCS	Radar Cross Section
RD	Range–Doppler
RFT	Radon Fourier Transform
RMSE	Root Mean Square Error
SKT	Second-Order Keystone Transform
SLC	Sidelobe Canceller
SNR	Signal-to-Noise Ratio
SoOPs	Signals of Opportunity
TMC	Time Migration Compensation
UAV	Uncrewed Aerial Vehicle
UKV	Unified Kinematic Vector
